# Combined inhibition of BCR-ABL1 and the proteasome as a potential novel therapeutic approach in BCR-ABL positive acute lymphoblastic leukemia

**DOI:** 10.1371/journal.pone.0268352

**Published:** 2022-10-04

**Authors:** Saskia Maletzke, Azam Salimi, Margherita Vieri, Kema Marlen Schroeder, Mirle Schemionek, Behzad Kharabi Masouleh, Tim H. Brümmendorf, Steffen Koschmieder, Iris Appelmann

**Affiliations:** 1 Department of Hematology, Oncology, Hemostaseology, and Stem Cell Transplantation, Faculty of Medicine, RWTH Aachen University, Aachen, Germany; 2 Center for Integrated Oncology Aachen Bonn Cologne Duesseldorf (CIO ABCD), Faculty of Medicine, RWTH Aachen University, Aachen, Germany; 3 Department of Palliative Medicine, Faculty of Medicine, RWTH Aachen University, Aachen, Germany; Universita degli Studi di Firenze, ITALY

## Abstract

Acute lymphoblastic leukemia (ALL) is a disease of lymphoid progenitor cells with an often aggressive course and is commonly caused by the BCR-ABL fusion gene t(9;22) in adults. This fusion gene encodes a constitutively active tyrosine kinase that can be effectively inhibited by tyrosine kinase inhibitors (TKIs), with imatinib being the paradigmatic agent of this class. However, BCR-ABL+ ALL cells rapidly develop mutations against many of the available TKIs, and consecutive disease relapse still results in an overall unfavorable prognosis for patients with this disease. To date, allogeneic stem cell transplantation is the only known curative therapeutic option for the mostly elderly patients with BCR-ABL+ ALL. The discrepancy between the limited therapeutic armamentarium and the growing therapeutic need in an aging population is therefore a reason to test drug combinations against BCR-ABL+ ALL. In this study, we demonstrate that the combination of TKIs with proteasome inhibitors efficiently and under certain conditions synergistically exerts cytotoxic effects in BCR-ABL+ ALL cells in vitro with respect to the induction of apoptosis. Both sole and combined treatment of BCR-ABL+ ALL with the proteasome inhibitors bortezomib and ixazomib, respectively, and TKI causes a significantly greater reduction in cell viability than TKI treatment alone in both BCR-ABL+ cell lines TOM-1 and BV-173. In BV-173 cells, we observed a significant reduction in cell viability to only 1.26%±0.46% with bortezomib treatment and 1.57±0.7% with combination treatment, whereas cells treated with dasatinib alone still had a viable percentage of 40.58±2.6%. Similar results were obtained when ixazomib was applied to both cell lines, and apoptosis was induced in both cases (93.36%±2.7% apoptotic BV-173 cells when treated with ixazomib and TKI). The combination of TKI and proteasome inhibitor is efficient in vitro, potentially expanding the spectrum of therapeutic options for patients with BCR-ABL+ ALL.

## Introduction

Acute lymphoblastic leukemia (ALL) is the most common malignancy in childhood and shows a second incidence peak in patients above 55 years of age [1]. With life expectancy steadily rising in many parts of the world, the incidence and prevalence of leukemia in adults increases. Therefore, specific and improved therapeutic options for adults and particularly the elderly are needed.

In 80% of patients with ALL, genetic aberrations can be found in the leukemic cells [2, 3], the most common lesion being the Philadelphia chromosome with its translocation t(9;22) accountable consecutively for the most common genetically defined subtype of adult ALL. Between 20 to 30% of all adult ALL patients and more than 50% of patients in the age group above 60 years carry this lesion associated with a dismal prognosis [4, 5]. The reciprocal translocation between the ABL-1 oncogene on chromosome 9 and the BCR gene on chromosome 22 results in a fusion gene which encodes the constitutively active oncogenic tyrosine kinase BCR-ABL [6, 7]. The current management in elderly patients with a BCR-ABL+ ALL comprises the use of a tyrosine kinase inhibitor (TKI) with optional chemotherapeutic treatment, depending on patient fitness and comorbidities. This is–if patient age and comorbidities allow- followed by an allogeneic stem cell transplantation (allo-SCT) which is still the only curative option for this dreadful disease [8]. Due to severe side effects, stem cell transplantation is often limited to patients below 65 years of age [1]. Addition of imatinib to chemotherapy improved patient prognosis and led to an overall survival of about 20 months despite the usually rapid development of resistance-conferring BCR-ABL mutations in Ph+ ALL [9]. Therefore, the second generation TKI dasatinib, which is able to bind the active as well as the inactive domain of the BCR-ABL kinase and overcomes a broad spectrum of mutations conferring clinical resistance to TKIs, is now an established therapeutic alternative to imatinib [10]. Dasatinib was successfully combined with the chemotherapy regimen CVAD by the EWALL group on the one hand leading to a 5-year overall survival (OS) of 36% of patients but simultaneously leading to considerable toxicity and consequently to an abandonment of this regime [7, 11]. In addition, single imatinib treatment was superior to the combination of imatinib and CVAD in elderly patients with Ph+ ALL, resulting in higher CR rates and less toxicity than induction chemotherapy [12]. Despite these improvements, the prognosis of patients with Ph+ ALL still remains poor [13]. The discrepancy between the growing therapeutic need in an aging population and the limited therapeutic options requires novel combined targeted therapeutic approaches that delay the development of resistance-conferring mutations and thereby lead to prolonged survival with a confined toxicity compared to conventional treatment regimens.

Our work investigates the combination of dasatinib with proteasome inhibitors as a prospective treatment option in Ph+ ALL in adults. ALL cells are characterized by an accelerated cell growth and proliferation and this increased proliferation, among other effects, causes a significant strain on the ubiquitin proteasome pathway [14]. The inhibition of the ubiquitin proteasome system (UPS) in addition to inhibiting the BCR-ABL kinase hence appeared to be a reasonable strategy against this leukemia subtype and led to our present study. Furthermore, it has been observed that inhibition of the unfolded protein response (UPR) is induced as a cell stress response in ALL and that it is an important point of action in BCR-ABL+ ALL [15]. The gene *ERN1* encodes for the serine/threonine-protein kinase and endoribonuclease, a key sensor of the unfolded protein response and mediates the splicing of *XBP1* [16, 17]. *XBP1* operates as a transcription factor, generated by a nonconventional splicing reaction and intercepts protein homeostasis in the endoplasmic reticulum [18]. This UPR activation is caused by an accumulation of misfolded proteins and finally–in concerted action with other mechanisms—culminates in apoptosis of Ph+ ALL cells. When UPR inhibitors were combined with TKIs in BCR-ABL+ ALL as well as with proteasome inhibitors in multiple myeloma, a striking synergistic effect occurred in both diseases [19], (Salimi et al., unpublished data). Also, multiple studies have revealed an inhibitory effect on the UPR by proteasome inhibition [17], thereby contributing to our rationale.

The first-generation proteasome inhibitor bortezomib is an efficient treatment for multiple myeloma and mantle cell lymphoma [20, 21] and has a more favorable toxicity profile with equal response rates after subcutaneous application compared to intravenous application [22]. Additionally, the second-generation and orally bioavailable proteasome inhibitor ixazomib has been approved for second-line treatment of multiple myeloma and is currently being investigated as a first line treatment option in multiple clinical studies (clinicaltrials.gov, e.g. NCI Identifier 01850524). Ixazomib will broaden the orally applicable therapeutic armamentarium with all of its obvious benefits for the patient and particularly for the necessary treatment adherence [23]. Both proteasome inhibitors are already approved by the FDA for multiple myeloma and show a favorable toxicity profile with limited overlap thus rendering it promising to administer proteasome inhibitors together with TKIs [24].

Takahashi et al. showed an anti-leukemic effect of bortezomib and carfilzomib on B-cell precursor ALL cell lines including Ph+ All cells with an IKZF1 deletion being a possible biomarker to predict proteasome inhibitor sensitivity [25].

Furthermore, Bertaina et al. showed that bortezomib combined with chemotherapy might be of potential advantage for relapsed ALL in childhood [26]. A case study by Dewar et al. reports a relapsed Ph+ ALL patient reaching a complete disease remission (CR) under combination treatment of bortezomib and the monoclonal anti-CD20 antibody rituximab [27]. While this clinical data was generated in relapsed disease, data on Ph+ ALL cells systematically treated with TKI and proteasome inhibition are missing to date. Our study shows encouraging effects when TKI and proteasome inhibitors are combined against Ph+ ALL cells in vitro and provides a rationale for further investigation in vivo and potentially also in a clinical setting.

## Material and methods

### Tissue culture

All human cell lines were originally obtained from DSMZ, Braunschweig, Germany, and were authenticated after passaging by Multiplexion®, Friedrichshafen, Germany. We used four human precursor B-cell ALL cell lines: TOM-1, BV-173, RS4;11 and REH (Table 1). They were cultured in Roswell Park Memorial Institute medium (RPMI-1640, Invitrogen®, Carlsbad, CA) with GlutaMAX® containing 10% fetal bovine serum, 100 IU ml^−1^ penicillin and 100 μg ml^−1^ streptomycin at 37°C in a humidified incubator with 5% CO_2_. The media was refreshed at least every third day. After 1–2 months, fresh cells were thawed. Viability was assessed by methylene blue exclusion staining before starting any experiment. Dasatinib and bortezomib were obtained from LC Laboratories® (Woburn, MA, USA), ixazomib was obtained from CaymanChemical® (via Biomol® GmbH, Hamburg, Germany). All drugs were dissolved in DMSO and stored at -20°C for further experiments.

**Table 1:** Characteristics and name of the used BCP-ALL cell lines.

used cell lines	
translocation	cell line
BCP-ALL (n = 4)	
Ph+ t (9;22) (n = 2)	TOM-1, BV-173
MLL+ t (4;11) (n = 1)	RS4;11
t (12;21) (n = 1)	REH

### Drugs and dosing

To determine the IC50 of bortezomib and ixazomib in both the TOM-1 and BV-173 cell lines we performed a cell proliferation assay using Cell Counting Kit-8 from Dojindo Molecular Technologies® (Munich, Germany).

Cells were seeded in a 96 well plate with 3x10^^5^ cells per well in a total volume of 200 μl. The cells were treated with increasing drug concentrations diluted in RPMI-1640 at day 0. Our dilutions ranged from 0.001 μM to 0.5 μM for both drugs. Each condition was assayed in six identical test-reactions as technical/biological replicates. At three different time points (24h, 48h and 72h), 10μl CCK8 were added to each well and after four hours of incubation the absorbance was measured at 450nm using a microplate reader (Thermo Fisher Scientific®, Waltham, MA, USA) After subtracting the media absorbance and normalizing the values, the results were analyzed via GraphPad® and transformed into a non-linear regression curve with the point of inflection representing the IC50. Dasatinib was used in a standard dosis of 50nM in each experiment.

### Flow cytometry

Cells were seeded in a six well plate in 3 ml of RPMI-1640 with 2x10^^6^ cells per condition. Drugs (dasatinib 50nM, different doses of bortezomib and ixazomib) were immediately added after seeding at day 0. A DMSO (Carl Roth®, Karlsruhe, Deutschland) control was included in each experimental set. 500μl were taken from every condition at each time point. Cells were washed with 3 ml phosphate buffered saline (PBS) and re-cultured with fresh medium and replenished drugs every three days. Cell viability was determined using propidium iodide (PI staining, 0.2μg/mL, Sigma-Aldrich®, St. Louis, MO, USA) as a cell death marker binding to double stranded DNA. After washing with 1 ml PBS at 400x g for 5 minutes and 20°C, cells were resuspended in 500μl PBS + 0.1% PI and analyzed via fluorescence assisted cell sorting (FACS). To quantify the fraction of apoptotic cells, we performed an apoptosis assay using annexin V binding buffer (0.1 M HEPES, 1.4 M NaCl, 25mM CaCl_2_ in H_2_O) instead of PBS. Cells were stained with an annexin-V-APC antibody (BD Biosciences®) and the aforementioned PI solution (2μg/mL). The annexin V-/PI- population represents the viable cells whereas annexin V+/PI- cells undergo early apoptosis. Dead or necrotic cells can be found in the annexin V+/PI+ population.

For cell cycle analysis, cells were seeded as described above. At every time point, 0.5x10^6^ cells were resuspended in saline-GM solution (Glucose 1.1g/l, NaCl 8g/l, KCl 0.4g/l, Na2HPO4.2H20 0.2g/l, KH2PO4 0.15g/l and EDTA 0.2g/l) and fixed using 90% ethanol. After washing with PBS + 5% FBS, the cells were stained for one hour in a solution of PI at a concentration of 10μg/mL in H_2_O with the addition of 25μg/mL Rnase (Sigma-Aldrich®, St. Louis, MO, USA). FACS reading was then performed. The entire procedure was carried out at 4°C. To investigate cell proliferation, we used a CFSE proliferation assay (CFSE solution: Sigma-Aldrich®, St. Louis, MO, USA).

At day 0, cells were incubated with CFSE solution for 15 min at room temperature and protected from light, media was added for another 5 minutes and then the cells were washed twice with PBS. Afterwards the cells were treated with the mentioned drugs and seeded in a six well plate with fresh media. At every time point 500μl were measured via FACS. All FACS data were analyzed via FlowJo®.

### Western blotting

Cells were seeded at 3x10^^6^ cell per well in a six well plate for 16 h. After harvest, cells were washed twice with PBS and lysed in RIPA buffer (50 mM TRIS, 150 mM NaCl, 1mM EDTA, 1% Triton-X, 15% Glycerol, 0,5% sodium desoxycholate in H_2_O) supplemented with protease inhibitors mix (Sigma®-Aldrich, St. Louis, MO, USA), sodium orthovanadate 1mM (Sigma®-Aldrich, St. Louis, MO, USA), sodium fluoride 20mM (Sigma®-Aldrich, St. Louis, MO, USA) and β-glycerophosphate 1mM (Sigma®-Aldrich, St. Louis, MO, USA). After 30 minutes of incubation on ice and centrifugation at 17,000 × g for 15 minutes at 4°C, the protein concentration was measured by Bradford assay (Bio-Rad®, Hercules, CA, USA) using a nanodrop spectrophotometer. If not used immediately, proteins were frozen at -80°C and stored for up to two weeks. 40 μg of protein per sample plus RIPA buffer and Laemmli buffer 1x (4x: 240 mM Tris pH 6.8, 40% glycerol, 8% SDS, 0.001% bromophenol blue, β-mercaptoethanol 5%) were loaded on 10% polyacrylamide gels casted with FastCast kits (BioRad®, Hercules, CA, USA). Samples were transferred to the gels in sodium dodecyl sulfate (SDS) running buffer (30.3 g tris base, pH 8.3, 144 g glycine, 10 g SDS) and ran at 30mA for 40 minutes/hours. Afterwards the proteins were transferred to a PVDF membrane using transfer buffer (3 g tris base, 14.4 g glycine, 10% methanol) at 350mA for 3h. After blocking, the membranes were incubated with the primary antibody of interest overnight at 4°C. Before incubating with the secondary antibody, the membranes were washed with TBS-T buffer, pH 7.4 (15 mM Tris-HCL, 150 mM NaCl) plus 0.05% IGEPAL® CA-630 (Sigma®-Aldrich, St. Louis, MO, USA) three times and after incubating the membranes were washed again. For protein detection PCA-ECL solution (100 mM Tris-HCL, pH 8.8, 2.5 mM luminol, 0.198 mM) p-coumaric acid and 0.2% v/v hydrogen peroxide (Sigma®-Aldrich, St. Louis, MO, USA) was used and light emission was detected by using a Fusion SL imaging system (Vilber®, Eberhardzell, Germany). The primary antibodies used were: Bcl-2 (50E3), BAX (D2E11), BIM (C34C5), PARP (Polyclonal #9542), Cell Signaling Technology®, Frankfurt, Germany) anti betaActin (Polyclonal #ab8227, Abcam®, Cambridge, UK). All primary antibodies were diluted 1:1000. The secondary antibody is Anti-Rabbit Immunoglobulins (DAKO®/Aligent ®, Santa Clara, CA, USA).

Densitometry analysis was performed with ImageJ® software.

### Quantitative RT-PCR and genomic PCR

Total RNA from cells was extracted from the Ph+ ALL cells using Trizol reagent (Ambion®/ Thermo Fisher Scientific®, Waltham, MA, USA) following by chloroform extraction after 24h of treatment. cDNA was generated using random hexamers and the M-MLV Reverse Transcriptase (Invitrogen® Carlsbad, CA, USA). Quantitative real-time PCR was performed with the SYBR Green mix (Invitrogen®, Carlsbad, CA, USA) and the ABI7500 fast real-time PCR system (Applied Biosystems®, Foster City, CA, USA) according to standard PCR conditions. The primers used for quantitative RT-PCR are *TRAIL* and *ERN1* (Eurofins,® Brussels, Belgium). The housekeeping gene *COX6b* (Eurofins®, Brussels, Belgium) represents the internal reference gene. The used primers and sequences are shown in Table 2. Data were analysed using the ΔΔCt method to calculate the relative quantification. Changes in gene expression were detected after 24h hours of treatment. The cells were treated with dasatinib 50nM, bortezomib 5nM, ixazomib 25nM and the combination between TKI and proteasome inhibitor.

**Table 2:** Primers and sequences used.

Name	manufacturer	sequence
*COX6b*	eurofins genomics	F–AACTACAAGACCGCCCCTTT
		R–GCAGCCAGTTGAGATCTTCC
*XBP1s*	eurofins genomics	F–AACCAGGAGTTAAGACAGCGCTT
		R–CTGCACCCTCTGCGGACT
*ERN1*	eurofins genomics	F–GACCGGCAATTCCAGTACAT
		R–TTGGGCATGGATATGAGGAT
*NOXA*	eurofins genomics	F–CTCTTTCCTCCTCGCCACTT
		R–GAGTCCCCTCATGCAAGTTT
*TRAIL*	eurofins genomics	F–GCAGGAATTCAGGATCATGGCTATGATGG
		R–GCACGGATCCCAGGTCAGTTAGCCAACT

### Statistical analyses

Data were analysed with GraphPad® Prism (GraphPad Prism 5.00.288) using analysis of variance (ANOVA) and Bonferroni multiple comparison tests. A p value of less than 0.05 was considered statistically significant for all analyses (*,p<0.05, **,p<0.01, ***,p<0.001).

### Coefficient of drug interaction (CDI)

To determine whether the tyrosine kinase inhibitor and the proteasome inhibitor act synergistically, we used the coefficient of drug interaction: *CDI* = *AB*/(*AxB*) (AB, relative cell viability of the combination; A or B, relative cell viability of the single agent groups). A CDI < 1 indicates a synergistic effect, a CDI = 1 indicates an additive effect and a CDI > 1 indicates an antagonistic effect.

## Results

### Treatment with proteasome inhibitors leads to growth arrest in Ph+ ALL cells

For a primary impression of the effects proteasome inhibitors have on BCR-ABL+ cells, we first determined the IC50 for bortezomib as described above. Bortezomib caused a strong decrease in viability in both the TOM-1 and the BV-173 cell line at a concentration of 5nM detected via CCK8 assay. At a higher dose (10nM), effects were so strong that no viable cells were left after only 24 hours of treatment (S1 Fig). A non-linear regression curve showed an IC50 for TOM-1 of 5nM and of 4.48nM for BV-173 on day 3 (S1 Fig and Table 2).

Next, we treated the cells with bortezomib again, tested the calculated IC50 concentrations and assayed cell viability on three consecutive days starting 24 hours after treatment initiation (results not shown). Consequently, we determined 5nM for TOM-1 and 6.25nM for BV-173 as most promising dose levels for our combination treatment with dasatinib. All following experiments were performed with these doses. Dasatinib was then applied at 50nM in all further combination treatments [28]. The drugs were solved in DMSO as described above, therefore DMSO was employed as a vehicle control in equal volume as the highest drug concentration. That was done in all following experiments. Both cell lines were treated with the IC50 of bortezomib and with bortezomib at a concentration of 4nM separately in order to potentially reveal potential additive effect between TKI and proteasome inhibitor with this lower concentration since both drugs by themselves already show a relatively high initial efficiency.

### Proteasome inhibitor treatment causes a significant reduction of viability in Ph+ ALL cell lines

**Fig 1:**
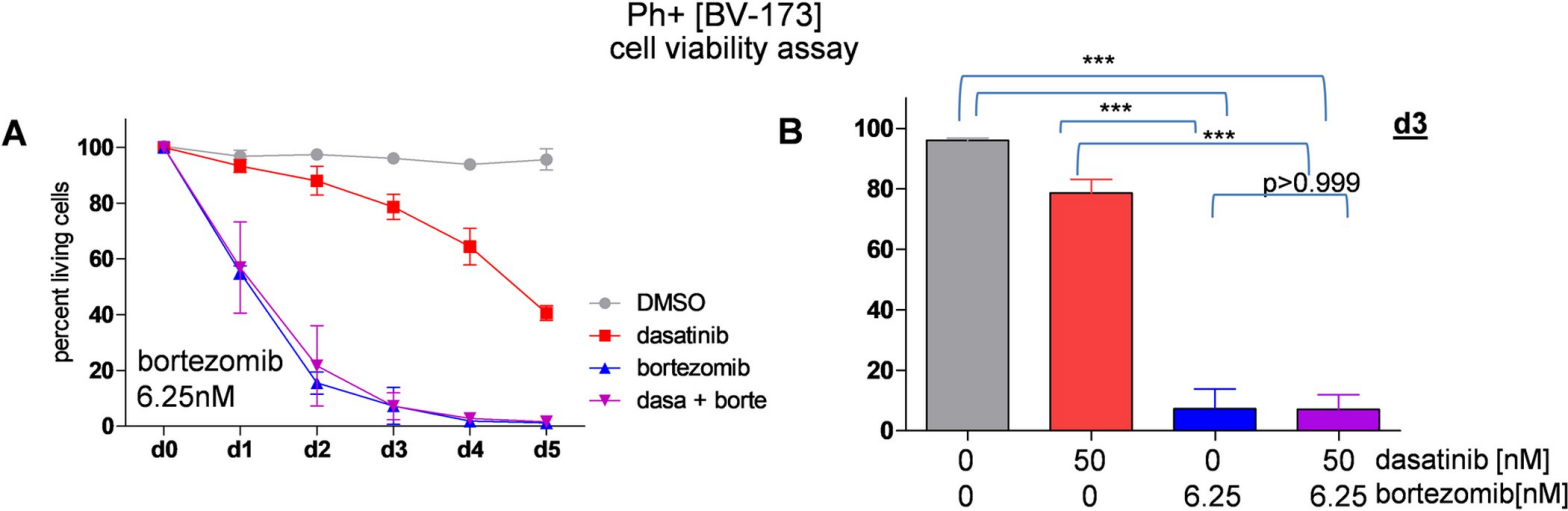
Combination treatment reduces cell viability and induces apoptosis in BV-173 cells. **(A)** Ph+ cells BV-173 were seeded with DMSO, dasatinib 50nM, bortezomib 6.25nM and the combination at day 0, drugs were replenished every third day, respectively. Viability was measured every 24h until day 5 using PI staining. **(B)** At day 3 cell viability was significantly reduced to only 7.27%±6.6% with bortezomib treatment and 7.16±4.8% with the combination treatment whereas the dasatinib only treated cells were 78.62±4.5% viable. For all analyses, p values were calculated by one-way ANOVA with Bonferroni multiple comparison test. A p value of less than 0.05 was considered statistically significant for all analyses. (*,p<0.05, **,p<0.01, ***,p<0.001), error bars = SD, n = 3/3).

Our two Ph+ ALL cell lines, TOM-1 und BV-173 differed only non-significantly in their reactions to our treatments but we nevertheless examined each cell line separately. The cells were treated with bortezomib and dasatinib for up to five consecutive days. The viability of BV-173 was significantly reduced after treatment with bortezomib at the dose of 6.25nM and in combination with dasatinib. After 24 hours of culture, 93.25±2.3% of the cells treated with single agent dasatinib were alive, whereas in the bortezomib treated cells only 55.13±2.42% of cells were viable. The combination treatment did also show a strong reduction of cell viability to 56.9±16.34% but was not able to further improve the effect on the leukemia cells (Fig 1A).

After three days of culture, cell viability was significantly reduced to only 7.27%±6.6% with bortezomib treatment and 7.16±4.8% with the combination treatment whereas the dasatinib only treated cells showed a viable fraction of still 78.62±4.5%. (Fig 1B) After five days the dasatinib only treated cells were still 40.58±2.6% viable, compared to only 1.57±0.7% viable cells in the comminated treatment (Fig 1A). This striking difference (p<0.001) between TKI and single proteasome inhibitor bortezomib as well as combined treatment is apparently mainly caused by proteasome inhibition, and no additive effect was detected by combining bortezomib with dasatinib in this particular cell line, and no CDI could be calculated. However, with regard to a potential later clinical investigation, it is important to note that there was no antagonism in the combination treatment and hence adding bortezomib to the current standard treatment might provide an efficient therapeutic option after further in vitro and in vivo investigation, especially when treatment is continued for longer periods of time.

In the TOM-1 line, we also detected a significant difference in the fraction of living cells between untreated and dasatinib-treated cells compared to cells treated with bortezomib or the combination of both drugs already at day 1. The untreated cells showed a viability of 97.22±4.8% whereas treatment with bortezomib (5nM) reduced the viability to 64.86±10.36% and the combination (dasatinib 50nM + bortezomib 5nM) treatment led to a viability of 61.64±4.87% on day 1 (S2A Fig).

Cell viability decreased to 73.49±10.2% after three days of dasatinib treatment while bortezomib treatment left only 17.78±13.7% of cells alive at this time point. The combination treatment with both drugs led to 12.32±8.9% of viable cells (S2B Fig). Hence, bortezomib alone as well as in combination with dasatinib causes a significant, namely 10- to 20-fold, difference in the viable cell fraction compared to untreated cells or cells treated with one of the current standard therapies (dasatinib). For bortezomib at a concentration of 5nM, we did also not observe a significant difference between single treatment compared to the combination of dasatinib and bortezomib in the TOM-1 cell line so that the CDI could not be calculated in this case (S2A Fig).

In the TOM-1 cells we also tested bortezomib 4nM as a lower concentration. Despite the fact that we were not able to compare both dose levels side by side for TOM-1 cells, we did observe that the lower dose level of 4nM bortezomib might cause an additive or even synergistic effect when combined with dasatinib. Especially after five days of treatment, we observed a significant difference between the viability of bortezomib-treated cells (61.01±25,3%) and the dasatinib-treated cells (43.21±10.97%) compared to the cells undergoing the combination treatment with both drugs (21.97±12.00%). The calculated CDI was 0.84, hence showing that both drugs tend to act synergistically in this particular cellular context (S2C+S2D Fig). These results are shown in the S2 Fig and need to be examined in detail in further experiments. We also treated the BV-173 cell line with the lower dose of bortezomib (4nM), either as a single agent or combined with dasatinib (50nM). Interestingly, we were not able to observe a synergistic effect of dasatinib and proteasome inhibition in these cells, because the effect of dasatinib seems to outweigh the effect of bortezomib (S3 Fig).

### Proteasome inhibitor treatment causes apoptosis in Ph+ ALL cells

**Fig 2:**
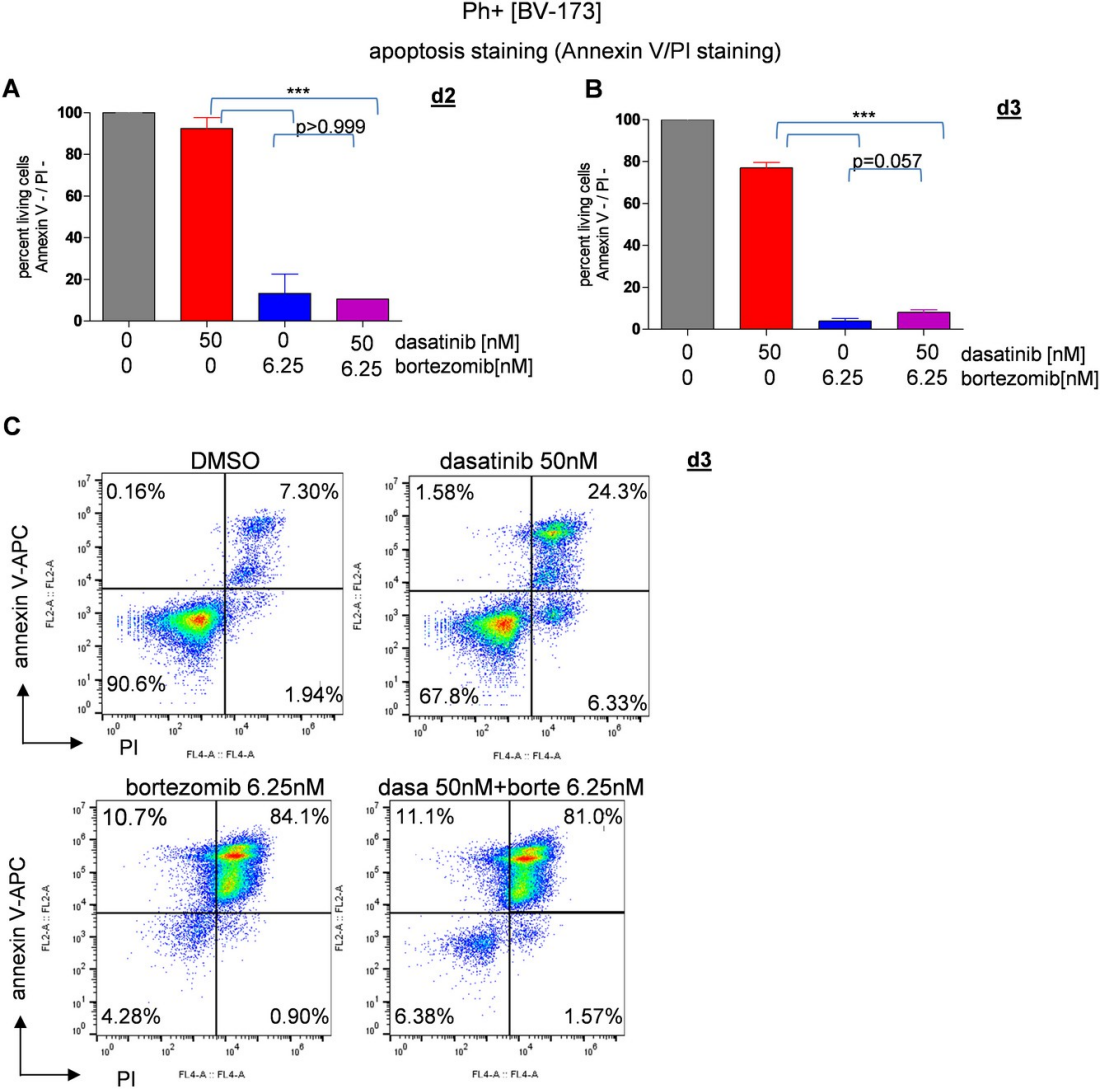
Apoptosis induction by combination of bortezomib and dasatinib in BV-173 measured via annexin V / PI staining. **(A+B)** Ph+ cells BV-173 treated with DMSO, dasatinib 50nM, bortezomib 6.25nM. Apoptosis assay was separately performed at day 2 and day 3 for both cell lines, only d3 is shown in the graphics. Bortezomib as well as combination treatment leads to apoptosis. At day 3 only 8.1±1.22% of the combinated treated cells were non apoptotic (Annexin V-/PI-). We detected no significant difference between bortezomib only and dasatinib + bortezomib treatment (p>0.999 at day 2 and p = 0.057 at day 3). Even though only two sets of experiments have been performed, we argue that a third pass would not have changed the set of results. The reader should please be aware of the n = 2 here. **(C)** With the combination treatment of dasatinib and bortezomib, 81.0% of BV-173 cells are found in late apoptosis at day 3 (annexin V+/PI+). For all analyses, p values were calculated by one-way ANOVA with Bonferroni multiple comparison test. A p value of less than 0.05 was considered statistically significant for all analyses. (*,p<0.05, **,p<0.01, ***,p<0.001), error bars = SD, n = 2/2).

To further elucidate how bortezomib actually affects the BCR-ABL+ cells, we performed CFSE and cell cycle assays but did not observe a significant impact of the different treatments on cell proliferation and cell cycle (S5 Fig). These assays were hence performed only ones and we then focussed on apoptosis as a potential predominant effect of proteasome inhibitor treatment in the context of proteasome inhibitor treatment in Ph+ ALL. For reason of non-significance and non-relevance the CFSE data are not shown here. To examine the apoptosis caused by TKI and proteasome inhibitor treatment an apoptosis assay with annexin V and PI was performed as described above. On day 3, 67.8% of the dasatinib-treated BV-173 cells were non-apoptotic (annexin V-/PI-) compared to 24.3% of apoptotic (annexin V+/PI+) cells. In contrast, bortezomib treatment caused massive cell death already at day 2 with only 13.2% BV-173 cells being alive (annexin V-/PI-) (Fig 2A+2C). The assay was repeated after three consecutive days of culture with the aforementioned treatment regimen (Fig 2). 84.1% of the BV-173 cells treated with bortezomib then being annexin V+/PI+ at day 3. Also, the combined treatment of dasatinib and bortezomib (6.25nM) led to a significant decrease in cell viability with 81.0% of cells in the combination treatment being annexin V+/PI+ (Fig 2C).

We also observed very similar results for the TOM-1 cell line (S4B Fig). Taken together, bortezomib treatment alone and in combination with TKI was highly efficient in inducing apoptosis of BCR-ABL1+ ALL cells in vitro.

### Ixazomib treatment causes a proliferation arrest in BV-173 and TOM-1

Even though bortezomib as a first-generation proteasome inhibitor already showed such strong effects on Ph+ ALL cell lines, we also wanted to investigate how the second-generation proteasome inhibitor ixazomib and especially its combination with TKI affects Ph+ ALL cells. Therefore, we first defined the IC50 of ixazomib performing a CCK8 staining assay as described for bortezomib. We observed an arrest of proliferation in TOM-1 and BV-173 cells with ixazomib at a concentration of 50nM (non-linear regression curve). The IC50 at day 3 for TOM-1 was 57.2 nM and 41.2nM for BV-173 (S6 Fig). We hence treated the cells with ixazomib applying these calculated IC50 values and measured cell viability. Afterwards, we established ixazomib at concentrations of 25nM and 30nM for BV-173 and 25nM for TOM-1 as most promising for a combination treatment with dasatinib in order to be able to see effects of the combination treatments more clearly. Other concentrations were also tested, e.g. 20nM, 30nM and 35nM. They led to comparable results as described above and are therefore not shown in detail. All dose levels were tested side by side.

### Recovery of ixazomib-treated cells results in a time-dependent synergism with TKI

**Fig 3:**
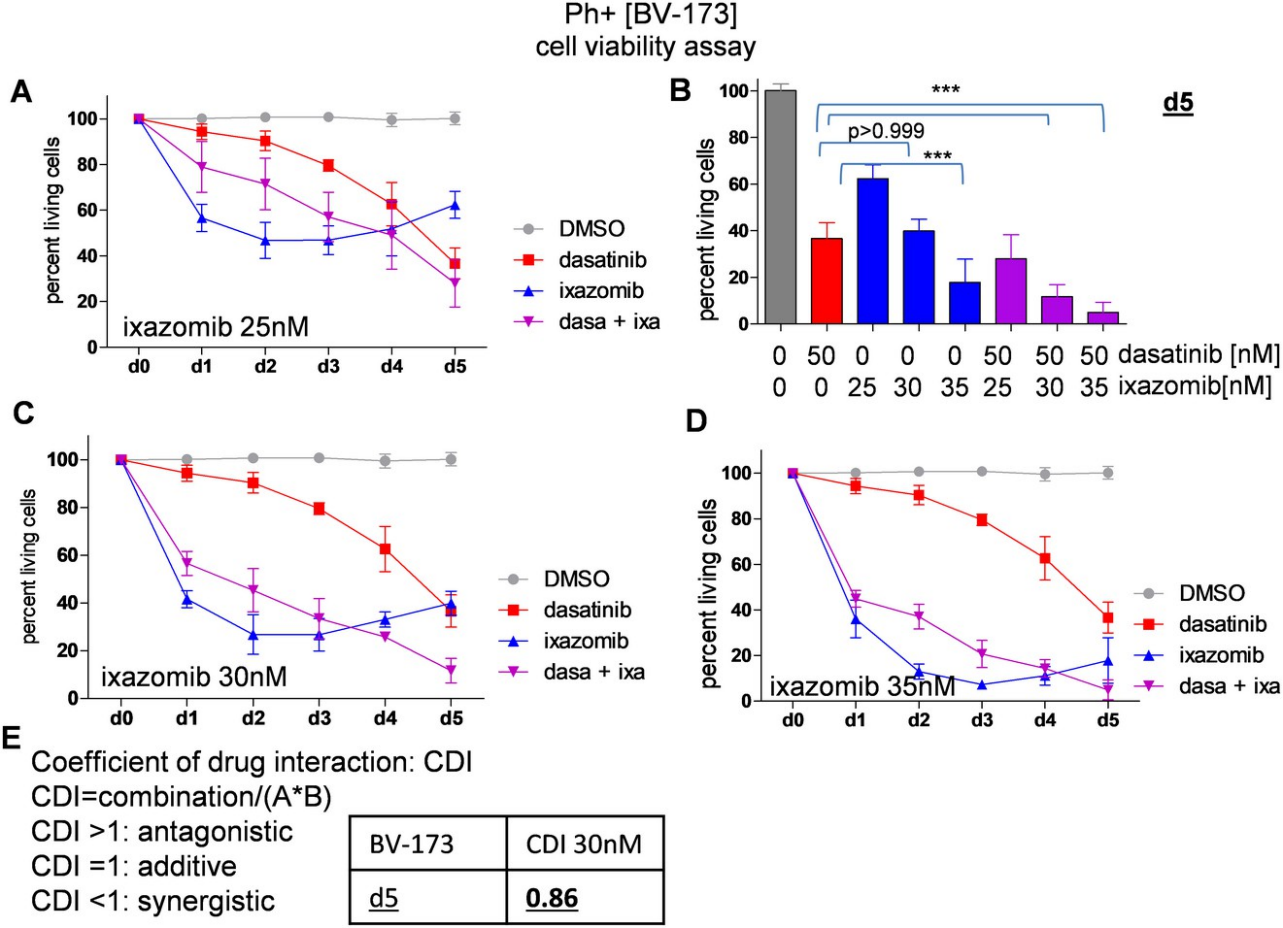
Combination treatment reduces cell viability and induces apoptosis in BV-173. Ph+ cells BV-173 were seeded with DMSO, dasatinib 50nM, ixazomib 25nM, 30nM, 35nM and the respective combination at day 0, drugs were replenished every third day. Viability was measured every 24h up to day 5 by PI staining assay. Recovery of cell viability was observed with ixazomib treatment despite drug replenishment for all ixazomib concentrations. **(A)** Treatment with dasatinib 50nM + ixazomib 25nM leads to a continuous reduction of the viability of BV-173, ixazomib only treated cells recover after day 3. **(B+C)** Single and combination treatment ixazomib 30nM: The treatment with ixazomib 30nM induced a strong reduction of viability to only 26.85±8.29% of viable cells after two days of culture in the BV-173. Combination treatment of dasatinib and ixazomib 30nM led to a continuous reduction of cell viability ending up with only 11.71±5.1% of viable cells on day 5. **(D)** Treatment with ixazomib 35nM reduces cell viability in single or combination treatment. **(E)** dasatinib 50nM + ixazomib 30nM: The calculated CDI was 0.86 on day 5, representing a synergistic effect of these two drugs in combination. (p values were calculated by one-way ANOVA with Bonferroni multiple comparison test. *,p<0.05, **,p<0.01, ***,p<0.001, error bars = SD, n = 3 for each treatment).

We cultured the cells with either dasatinib, again at 50nM, or ixazomib at 25nM or 30nM, respectively, or the combination of both drugs. The treatment with ixazomib 30nM induced a strong reduction of viability to only 26.85 ± 8.29% of viable cells after two days of culture in the BV-173 (Fig 3C). Much to our surprise, the cells treated with ixazomib alone began to recover after day 3 despite replenishment of the drug in the culture medium. At day 4, we detected 33.1±3.1% of viable cells, and the viable cell fraction even increased to 39.89±1.68% on day 5, still with drug replenishment after each cell suspension dilution. In contrast, the combination treatment of dasatinib and ixazomib 30nM led to a continuous reduction of cell viability ending up with only 11.71±5.1% of viable cells on d 5. The calculated CDI was 0.86 on day 5, representing a synergistic effect of these two drugs in combination (Fig 3E).

Our BV-173 Ph+ ALL cells treated with ixazomib at 25nM also showed a significant difference in viability compared to dasatinib single treatment on day 2 with 46.79±7.8% of viable cells in the ixazomib mono-therapy group compared to still 90.37±1.41% of viable dasatinib treated cells (Fig 3A and 3B). Again, a recovery of cell viability in the ixazomib only treated cells was observed after three days of drug exposure, with a viability of 62.35±5.89% at day 5 (Fig 3A and 3B). At this time point the TKI effect outweighed the effects of proteasome inhibition and no benefit of combination treatment was detected. (36.65±6.76% cells viable with dasatinib alone and 27.97±10.3% viable cells when combined with ixazomib) (Fig 3A and 3B). The cells were treated with 35nM ixazomib as well, showing similar results (Fig 3D).

The TOM-1 cells were treated with ixazomib and dasatinib in the same manner. We obtained comparable results for 25nM and 30nM ixazomib in the PI Staining for TOM-1. For this reason, only the results for 25nM are described below. The viability of TOM-1 was significantly reduced by ixazomib single treatment as well as in combination with dasatinib (S7 Fig). With ixazomib at a concentration of 25nM, viability was reduced to 9.8±4.45% in single and 6.2±6.5% in combination treatment at day 5 (S7B Fig). Since we did not observe a significant difference between both conditions a CDI could not be calculated. Ixazomib alone and combined with dasatinib showed a significantly stronger effect (p<0.001) on cell viability than single treatment with dasatinib: 41.51±14.22 of the dasatinib treated cells were still viable at day 5. We did not observe a significant recovery phenomenon of the cells in this specific context (S7A Fig).

Furthermore, for both cell lines we detected a dose-dependent effect with proteasome inhibitor treatment. For the BV-173 cells at day 5, the reduction of cell viability caused by 30nM ixazomib was significantly stronger (***, p<0.001) than the decrease in viability caused by ixazomib 25nM. The same difference was be observed between the two combination treatments dasatinib + ixazomib 25nM and dasatinib + ixazomib 30nM (Fig 3B). As for all other experiments, dasatinib was applied at a concentration of 50nM. For the TOM-1 cells, a similar dose-dependent difference between the reduction of the cell viability caused by different ixazomib dose levels was observed.

**Fig 4:**
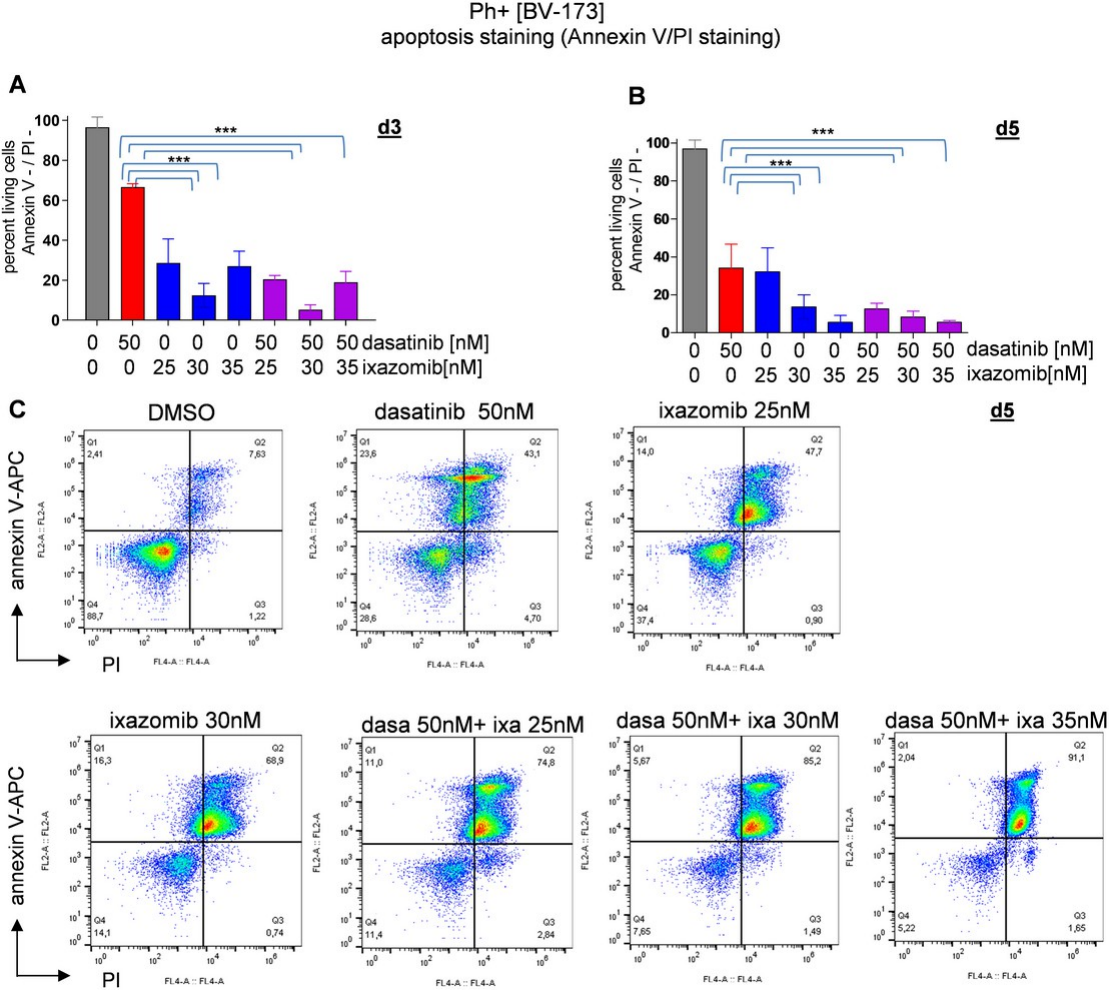
Comparison of apoptotic cell fraction in cells treated with TKI combined with bortezomib or ixazomib, respectively, measured by annexin V / PI staining. Ph+ cells BV-173 were seeded with DMSO and ixazomib at day 0, apoptosis assay was performed at day 3 and day 5. Treatment with ixazomib causes apoptosis in Ph+ ALL cells, detected at day 3 and day 5. **(A)** At day 3 26.96±7.6% of the cells treated with ixazomib 25nM + dasatinib were non apoptotic (annexin V-/PI-). **(B)** At day 3 12.56±3.1% of the cells treated with ixazomib 25nM + dasatinib were non apoptotic (annexin V-/PI-). In the graphics only day 5 is shown. **(C)** 74.8% in late apoptosis at day 5 (annexin V+/PI+) with the dual treatment (ixazomib 25nM + dasatinib) and 2.84% in early apoptosis. 85.2% in late apoptosis at day 5 with dasatinib and ixazomib 30nM. For all analyses, p values were calculated by one-way ANOVA with Bonferroni multiple comparison test. A p value of less than 0.05 was considered statistically significant for all analyses. (*,p<0.05, **,p<0.01, ***,p<0.001), error bars = SD, n = 3/3).

We next performed an apoptosis assay on day 3 and day 5 for both cell lines after ixazomib treatment. On day 3, 66.68±1.62% of the BV-173 cells treated with dasatinib only were viable compared to 12.44±6.0% for ixazomib 30nM and 20.56±1.83% for the combination treatment (Fig 4A). On day 5, an apoptosis assay performed with the ixazomib 30nM treated BV-173 cells showed 13.67±6.3% viable cells in comparison to 6.64±2.7% viable cells with the combination treatment (Fig 4B). Comparable results were obtained for the TOM-1 cells with these cells also undergoing apoptosis with proteasome inhibitor monotherapy (S8 Fig).

### TKI and proteasome inhibitor dual treatment leads to apoptosis in Ph+ ALL cells

**Fig 5:**
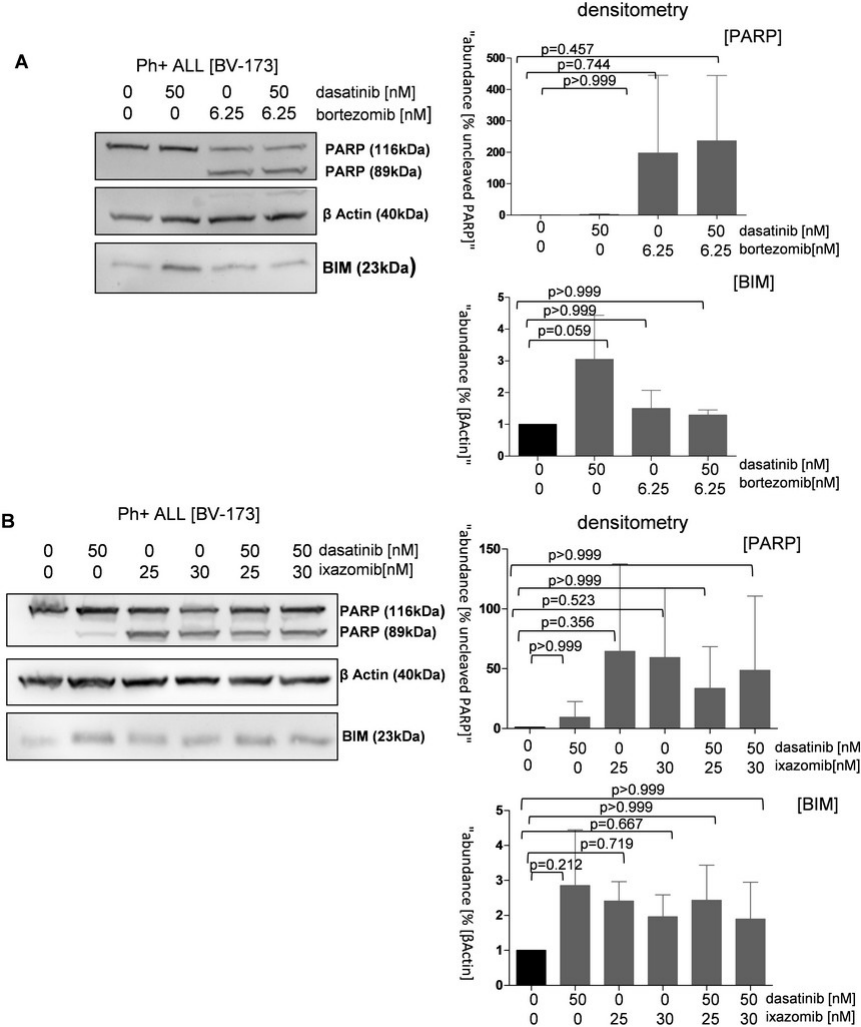
Expression of cleaved PARP and BIM as proapoptotic markers explain additive effects. Cells were treated for 16 hours with dasatinib 50nM, proteasome inhibitor bortezomib and ixazomib and the combination of both drugs. Proteasome inhibitor treatment causes an upregulation of cleaved PARP as a marker of apoptosis in alle treated cells. TKI treatment upregulates proapoptotic BIM. Densitometry analysis was performed using ImageJ® software and also showed an upregulation of PARP-1 under proteasome inhibitor therapy and an increased expression of BIM by dasatinib administration. These results were not statistically significant. (p values were calculated by one-way ANOVA. *,p<0.05, **,p<0.01, ***,p<0.001, error bars = SD, n = 3 for each treatment.

To quantify the apoptotic cell fraction in the BV-173 and TOM-1 cell lines undergoing proteasome inhibitor and TKI treatment we performed Western blotting for apoptotic markers, among them cleaved Poly [ADP-ribose] polymerase 1 (PARP-1). This protein is cleaved by several caspases involved in programmed cell death [29]. In our Western blots, we detected PARP cleavage and an increase PARP expression in all proteasome inhibitor treated cells, supporting our hypothesis that proteasome inhibitors lead to apoptosis in Ph+ ALL cells. PARP cleavage was detected in single bortezomib and ixazomib treated cells in both cell lines as well as in the dually treated cells. In contrast, the dasatinib single and in the untreated cells did not show PARP-1 cleavage. Hence, apoptosis induced by proteasome inhibitor treatment in Ph+ ALL cells mediates the cleavage of the pro-apoptotic protein PARP-1. For the dasatinib treatment, we observed an upregulation of the pro-apoptotic member of the BCL-2 family BIM in all dasatinib treated cells. BIM is one of the BH3-only proteins mainly included in the intrinsic apoptotic pathway and located in the outer mitochondrial membrane [30, 31]. The expression of the proteins BIM and PARP-1 in the western blot were analyzed by densitometry, which also showed an upregulation of PARP-1 under proteasome inhibitor therapy and an increased expression of BIM by dasatinib administration. However, in the one way anova, these results were not statistically significant (Figs 5 and S9).

### qPCR detects increased expression of *ERN-1* und *TRAIL*

**Fig 6:**
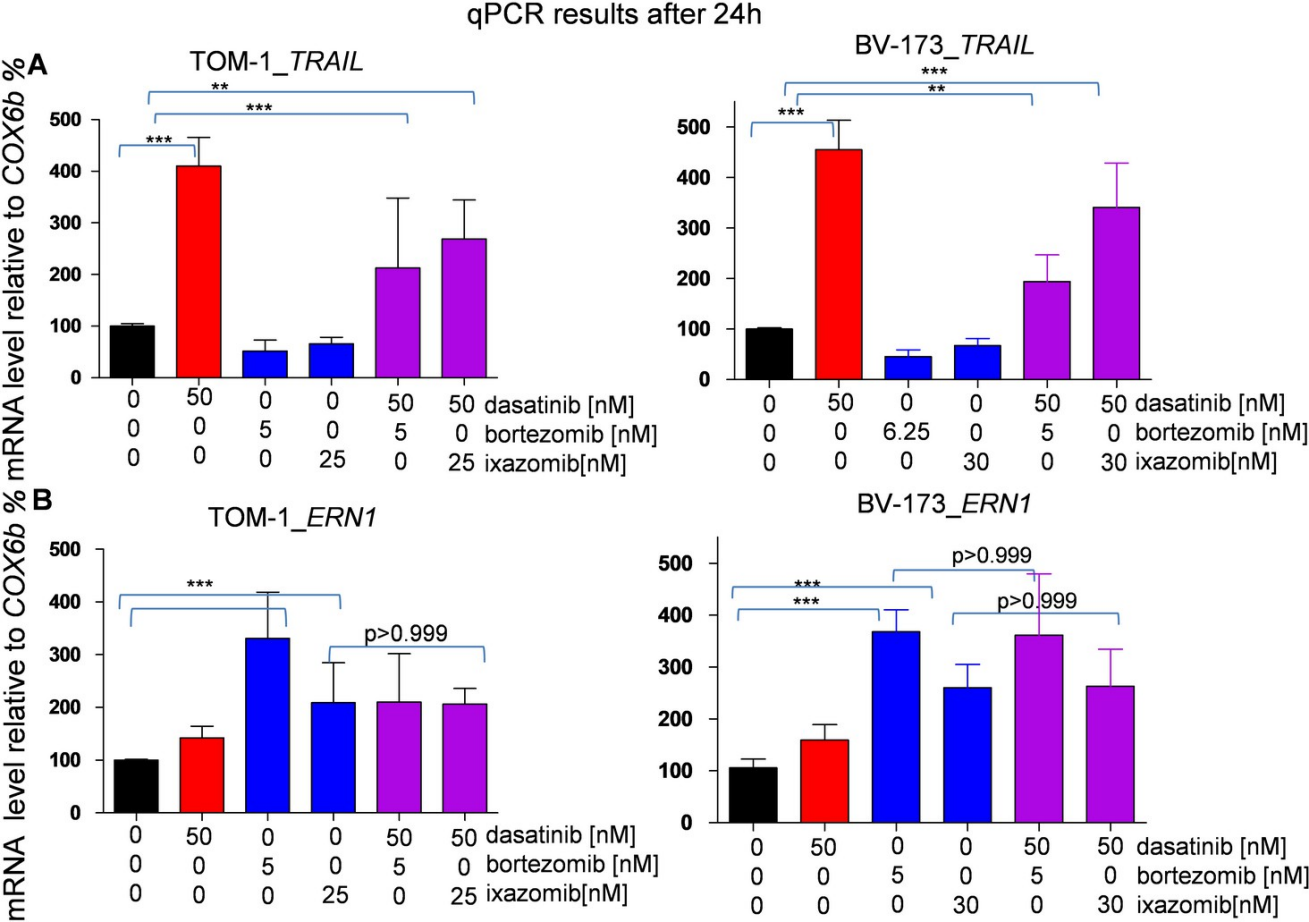
Dasatinib treatment causes *TRAIL* expression on the mRNA level and proteasome inhibitor treatment leads to *ERN1/XBP1* expression. Ph+ ALL cells TOM-1 and BV-173 were treated with dasatinib, proteasome inhibitor bortezomib or ixazomib and the combination of dasatinib and proteasome inhibitor. qPCR was performed after 24h of treatment. **(A)** Dasatinib induces a significant upregulation of *TRAIL* in all dasatinib treated cells for TOM-1 and BV-173. **(B)** A significant upregulation of *ERN1* in all proteasome inhibitor treated cells was detected via qPCR. No significant difference between ixazomib single treatment or combination treatment in terms of *ERN1* expression was observed. (p values were calculated by one-way ANOVA. *,p<0.05, **,p<0.01, ***,p<0.001, error bars = SD, n = 4 for each treatment).

Focusing on apoptosis caused by TKI and proteasome inhibitor treatment in Ph+ ALL cells, we also performed a two-step real time-qPCR and observed in both TOM-1 and BV-173 cells a significant (p<0.001,***) upregulation of proapoptotic *TRAIL* (tumor necrosis factor related apoptosis inducing ligand) in all dasatinib treated cells. The highest fold differences in expression compared to our housekeeping gene (*COX6b*) were detected for dasatinib 50nM single treatment. We detected a significant induction of *TRAIL* expression in the Ph+ ALL cells treated with the drug combination compared to untreated as well as proteasome inhibitor treated cells (Fig 6A).

In both cell lines the application of proteasome inhibitors led to a significantly higher (p<0.001 ***) *ERN1* expression compared to the untreated or dasatinib treated cells. The *ERN1* expression detectable in the BV-173 cells treated with bortezomib or ixazomib is as high as the *ERN1* expression in the combined treated cells, highlighting the substantial role of proteasome inhibitor treatment in this context. For the TOM-1 cells treated with ixazomib or the combination of ixazomib and dasatinib we did not detect a significant difference in *ERN1* expression (Fig 6B).

### Treatment with proteasome inhibitors significantly reduces the viability in Ph- ALL cells

Finally, we wanted to know how proteasome inhibitor treatment affects Ph- ALL cells. We therefore treated the two Ph- cell lines REH and RS4;11 with the same proteasome inhibitor concentrations used before and performed a PI staining assay each day till day 4. The proteasome inhibitor treatment led to a more pronounced and significant reduction of the viability in both cell lines. After day 3, 14.84±9.18% of the REH cells treated with bortezomib 5nM were viable and only 21.01±7.43% of the cells treated with ixazomib treatment were alive (S10A Fig). For the RS4;11 cells, only 0.16±0.03% of the cells treated with bortezomib were viable. Similar effects were caused by ixazomib 30nM treatment: only 1±0.94% of the treated cells were alive at day 3 (S10B Fig). Proteasome inhibitor treatment causes striking cytotoxic effect also in Ph- ALL and especially the RS4;11 are very sensitive to this substance class. Detailed results are shown in S10 Fig.

## Discussion

In the present study, we systematically investigated the efficacy of proteasome inhibitors alone and in combination with TKIs in cell lines derived from patients with Ph+ ALL. Our data provide insight into how the combination of TKIs and proteasome inhibitors affects Ph+ ALL cells and suggest that combined inhibition of BCR-ABL kinase and proteasome is an efficient strategy for the treatment of Ph+ ALL leukemia. Recent studies have already demonstrated an antileukemic effect of bortezomib in pediatric B-cell precursor ALL in relapsed cases [32], and treatment with bortezomib has also been shown to have a tolerable nonhematologic toxicity profile [33]. From a pharmacotherapeutic perspective, it is reasonable and well justifiable to coadminister proteasome inhibitors with TKIs: Both proteasome inhibitors used in this study are FDA-approved for other hematologic malignancies [24], have a well-tolerated toxicity profile with limited overlap, and have no known pharmacologic interactions (http://reference.medscape.com/drug-interactionchecker) with TKIs. In TOM-1 and BV-173 cells, both single and combined proteasome inhibitor treatment caused a significantly greater reduction in the proportion of viable cells than TKI treatment alone, the latter being indeed the current standard therapy against Ph+ ALL in adults, either in combination with conventional chemotherapy, glucocorticoids, bispecific antibodies (e.g., blinatumomab), or their respective combinations [4, 9, 34]. Our results are consistent with those of Takahashi et al. who recently demonstrated an antileukemic effect of bortezomib on B-cell precursor ALL cell lines [25]. At lower doses, we observed a tendency for TKIs and bortezomib to possibly act additively in TOM-1, but so far this can only be considered a preliminary observation that requires further validation. Importantly, we did not observe antagonistic effects between TKIs and proteasome inhibitors in any of our experiments. In addition, we demonstrated that treatment with the orally bioavailable proteasome inhibitor ixazomib as a single agent or as a combination treatment, again together with TKIs, also led to a significant reduction in cell viability. In this context, it is important to emphasize that the synergistic effect between ixazomib and dasatinib that we observed in BV-173 cells was due to the recovery of cell viability during treatment with ixazomib alone after day 3. One interpretation of the observed recovery phenomenon could be that when treated with ixazomib alone, other mechanisms important for proteostasis, such as unfolded protein response (UPR) or autophagy, counteract the effect of the proteasome inhibitor. These mechanisms could be blocked by the combination treatment. Therefore, we plan to analyze the restored BV-173 cells in detail in future studies. However, the counteracting synergism with dasatinib abrogates the primary potent effects of ixazomib on cell viability, suggesting that this is a potentially beneficial combination for further preclinical and clinical investigation. In both cell lines, both single and combination treatment with ixazomib caused a dose-dependent and significantly greater reduction in cell viability than dasatinib alone. Since none of our combination treatments was antagonistic, but always had at least additive or even synergistic effects, further investigation of potential clinical applications after confirmation of our results in vivo is desirable, especially as an oral treatment regimen for elderly patients with Ph+ ALL.

Both proteasome inhibition and TKI treatment and the combination of both inhibitor classes mainly induced apoptosis in our Ph+ ALL cell lines, as shown by annexin V staining results. In protein analyses by Western blot, we therefore focused on the expression of the two pro-apoptotic markers BIM and PARP. In the TKI-treated cells, we observed an upregulation of BIM, which is also generally known to be increased in CML cells treated with dasatinib or imatinib [35], whereas we detected cleavage of PARP in cells treated with proteasome inhibitors and in the combination treatment. This is in agreement with results published by Esparis-Ogando et al. showing that treatment with bortezomib in plasma cell leukemia leads to PARP cleavage and upregulation of caspase 3, which are responsible for apoptosis in this specific cellular context [36]. In addition, we tested the extrinsic apoptotic pathway by qPCR and found significant induction of TRAIL expression by dasatinib alone and by combination treatment. TRAIL is primarily downregulated in Ph+ ALL, but its upregulation, and thus induction of cell death, has been described previously [6]. Taken together, our results indicate that apoptosis is induced via the extrinsic and intrinsic pathways in cells treated with the single agent and in the combination treatment and is a major mechanism for the observed reduction in cell viability. However, to clarify which apoptosis pathway plays a more dominant role when both TKIs and proteasome inhibitors are used together, further testing is needed.

The upregulation of *ERN-1* causing the splicing of *XBP1*, a key sensor of the UPR, in all proteasome inhibitor treated cells compared to untreated or dasatinib treated cells showed that proteasome inhibitor treatment caused significant ER-mediated cell stress. The UPR and the associated *ERN-1* expression are a complex multilayered system with a sensitive balance involved in apoptosis of Ph+ ALL caused by proteasome inhibitor treatment. The effect of ERN-1 upregulation and enhanced XBP1 splicing has also been observed in proteasome inhibitor treated multiple myeloma cells, ultimately contributing to cell death in this context [17]. In Ph+ ALL, *XBP1* is upregulated and patients with an induced expression of *XBP1* carry a poorer prognosis than those without an induction of this gene [15]. Our observations underline the importance of this cellular stress response in ALL and emphasize it as an inducible vulnerability of Ph+ ALL cells. Furthermore, the strong *ERN-1* gene expression caused by ixazomib as a single agent and by the dual treatment depicts that the TKI effect on this part of the cell is only minimal and implies that a major part of the response of Ph+ ALL cells to proteasome inhibitors is mediated through the UPR, enabling a synergistic effect between both drugs in BV-173.

Further investigation is needed to further substantiate our findings, including testing of both agents in a preclinical mouse model and in primary patient samples. The prognosis of Ph+ ALL patients carrying the T315I mutation is still worse than in patients without this mutation despite the efficacy of the new TKI ponatinib [37]. Establishing new therapeutic options is important to delay or even prevent the development of resistance-mediating mutations [38], and a follow-up of this study that then includes T315I-positive Ph+ ALL cell lines may show useful results in this regard [39, 40].

In conclusion, our results demonstrate a significant pro-apoptotic effect of proteasome inhibitors on Ph+ ALL cell lines. Treatment of BCR-ABL1-positive ALL with a combined therapeutic regimen that includes the current standard of care dasatinib together with proteasome inhibitors appears to be a promising approach but requires further preclinical and clinical validation.

## Supporting information

S1 Fig IC 50 determination for bortezomib for TOM-1 and BV-173 cells. Ph+ cells BV-173 (A+B) and TOM-1 (C+D) were seeded with DMSO and bortezomib at day 0. DMSO was employed as a vehicle control in equal volume as the drug volume added in combination treatment. CCK8 assay was performed at day 1,2 and 3. The IC50 is depicted in a non-linear regression curve with the point of inflections representing the respective IC50. The drug concentration ranged from 0.001μM to 0.5μM for both drugs. The concentrations and the corresponding logarithm to construct the regression curve are shown in Table 1. Table 2: We calculated an IC50 for TOM-1 of 5nM and of 4.48nM for BV-173 on day 3. (errobars = SD, n = 2 for each treatment).
(TIF)

S2 Fig Combination treatment reduces cell viability and induces apoptosis in TOM-1 cells and both drug combined do not antagonize each other. **(A)** Ph+ cells TOM-1 seeded with DMSO, dasatinib 50 nM, bortezomib 5 nM and the combination at day 0, drugs were replenished every third day. Viability was measured every 24h until day 5 by PI staining assay. The single as well as combined bortezomib treatment caused a massive reduction of the cell viability up to day 5. **(B)** Cell viability decreased to 73.49±10.2% after three days of dasatinib treatment while bortezomib treatment left only 17.78±13.7% of cells alive at this time point. **(C+D)** TOM-1 seeded with DMSO, dasatinib 50 nM, bortezomib 4 nM and the combination at day 0, drugs were replenished every third day. Viability was measured every 24h until day 5 by PI staining assay. At day 5 cell viability of the dasatinib treated cells decreased to 43.21±11%. Whereas the combination treatment led to only 21.97±12% viable cells. **(E)** Bortezomib 4nM: The calculated CDI was 0.84 on day 5, representing a synergistic effect of these two drugs in combination.(p values were calculated by one-way ANOVA with Bonferroni multiple comparison test. *,p<0.05, **,p<0.01, ***,p<0.001, error bars = SD, n = 3/3).
(TIF)

S3 Fig BV173 treatment with 4nM bortezomib. **(A+B)**: Ph+ cells BV173 were treated with DMSO, dasatinib 50nM, bortezomib 4nM and combination at day 0. Viability was measured every 24h to day 5 using PI staining assay. Dasatinib as well as the combination of dasatinib and bortezomib caused a reduction of the viability. On day 5 32.4±1.5% of the dasatinib treated cells were viable, compared to 31.5±2.3% viability in combinated treatment. No significant different between these treatment regimes. No antagonistic effect could be detected. The strong effect of dasatinib seems to outweighs the effect of lower dose bortezomib in combination treatment with 4nM bortezomib. (p values were calculated by one-way ANOVA with Bonferroni multiple comparison test. *,p<0.05, **,p<0.01, ***,p<0.001, error bars = SD, n = 3).
(TIF)

S4 Fig Apoptosis induction by combination of bortezomib and dasatinib in BV-173 and TOM-1 measured annexin V / PI staining assay. Ph+ cells TOM-1 were seeded with DMSO and bortezomib at day 0, apoptosis assay was separately performed on day 2 and day 3. Only day 3 is shown in graphics. **(A+B)** A significant proportion of TOM-1 cell treated with either bortezomib or the combinatorial treatment was apoptotic either at day 2 and day 3. **(C)** With the combination treatment of dasatinib and bortezomib, 86.4% of TOM-1 are detected in late apoptosis at day 3 (annexin V+/PI+). (p values were calculated by one-way ANOVA with Bonferroni multiple comparison test. *,p<0.05, **,p<0.01, ***,p<0.001, error bars = SD, n = 2/2).
(TIF)

S5 Fig Cell cycle assay detects no differences among treatment groups [n = 1]. There is no significant difference in cell cycle between treatment groups, especially no significant S-phase reduction in both cell lines TOM-1 and BV-173. (error bars = SD, n = 1 for each treatment).
(TIF)

S6 Fig IC50 determination ixazomib [n = 2] for TOM-1 and BV-173. Ph+ cells BV-173 and TOM-1 were treated with DMSO and bortezomib at day 0, CCK8 assay was performed at day 1,2 and 3. The IC50 is depicted in a non-linear regression curve with the point of inflections representing the respective IC50. The calculated IC50 values are shown in the table and ranged around 57nM for TOM-1 at d2 and 41.2nM for BV-173 at day 2. Errobars = SD, n = 2 for each treatment.
(TIF)

S7 Fig TOM-1: Combination treatment reduces cell viability and induces apoptosis. **(A+B)** Ph+ cells TOM-1 were treated with DMSO, dasatinib 50nM, ixazomib 25nM and the combination on day 0. Viability was measured every 24h to day 6 using PI staining assay. Drugs were replenished every third day. Ixazomib and combination treatment lead to a significant reduction of the viability. At day 5 only 6.2±2.9% of the combinated treated cells were viable. No synergistic activity between ixazomib and dasatinib as well as no antagonistic effect. (p values were calculated by one-way ANOVA with Bonferroni multiple comparison test. *,p<0.05, **,p<0.01, ***,p<0.001, error bars = SD, n = 3).
(TIF)

S8 Fig Comparison of apoptotic cell fraction in cells treated with TKI combined with bortezomib or ixazomib, respectively, measured by annexin V / PI staining. Ph+ cells TOM-1 were seeded with DMSO, dasatinib 50nM, ixazomib 25nM and the combination of both at day 0, apoptosis assay was performed at day 3 and day 5. **(A)** At day 3 13.4±9.7% of the cells treated with ixazomib 25nM + dasatinib were non apoptotic (annexin V-/PI-). **(B)** At day 5 8.1±5.8% of the cells treated with ixazomib 25nM + dasatinib were non apoptotic (annexin V-/PI-). In the graphics only day 5 is shown. **(C)** 80.7% in late apoptosis at day 5 (annexin V+/PI+) with the dual treatment (ixazomib 25nM + dasatinib) and 6.6% in early apoptosis. For all analyses, p values were calculated by one-way ANOVA with Bonferroni multiple comparison test. A p value of less than 0.05 was considered statistically significant for all analyses. (*,p<0.05, **,p<0.01, ***,p<0.001), error bars = SD, n = 2/2).
(TIF)

S9 Fig Expression of apoptotic markers cleaved PARP and BIM in response to treatment with TKI and proteasome inhibitors. TOM-1 cell were treated for 16 hours with dasatinib 50nM, proteasome inhibitor bortezomib and ixazomib and the combination of both drugs. Proteasome inhibitor treatment causes an upregulation of cleaved PARP in all treated cells. TKI treatment upregulates proapoptotic BIM. Densitometry analysis was performed using ImageJ® software and also showed an upregulation of PARP-1 under proteasome inhibitor therapy and an increased expression of BIM by dasatinib administration. These results were not statistically significant. (p values were calculated by one-way ANOVA. *,p<0.05, **,p<0.01, ***,p<0.001, error bars = SD, n = 2/3/3/3).
(TIF)

S10 Fig Ph negative ALL cell lines REH and RS4;11 respond to proteasome inhibitor treatment with a reduction of viable cells. Ph- ALL cells REH and RS4;11 were seeded with DMSO, bortezomib 4nM, 5nM, 6.25nM and ixazomib 25nM, 30nM at day 0, drugs were replenished every third day. Viability was measured every 24h to day 3/4 using PI staining assay. Treatment with bortezomib and ixazomib causes a significant (***) reduction of viability in the two Ph- ALL cell lines REH and RS4;11. At d314.84±9.18% of the REH cells treated with bortezomib 5nM were viable and only 21.01±7.43% treated with ixazomib treatment were alive. For the RS4;11 cells, only 0.16±0.03% of the cells treated with bortezomib were viable at day 3. Similar effects were caused by ixazomib 30nM treatment: only 1±0.94% of the treated cells were alive at day 3. (p values were calculated by one-way ANOVA with Bonferroni multiple comparison test. *,p<0.05, **,p<0.01, ***,p<0.001, error bars = SD, n = 3 for each treatment).
(TIF)

S1 Raw images (PDF)
