# Supplementary figures and images for: Combined inhibition of BCR-ABL1 and the proteasome as a potential novel therapeutic approach in BCR-ABL positive acute lymphoblastic leukemia
Source: PLoS One. 2022 Oct 4;17(10):e0268352. doi: 10.1371/journal.pone.0268352 (PMC9531817; doi:10.1371/journal.pone.0268352)

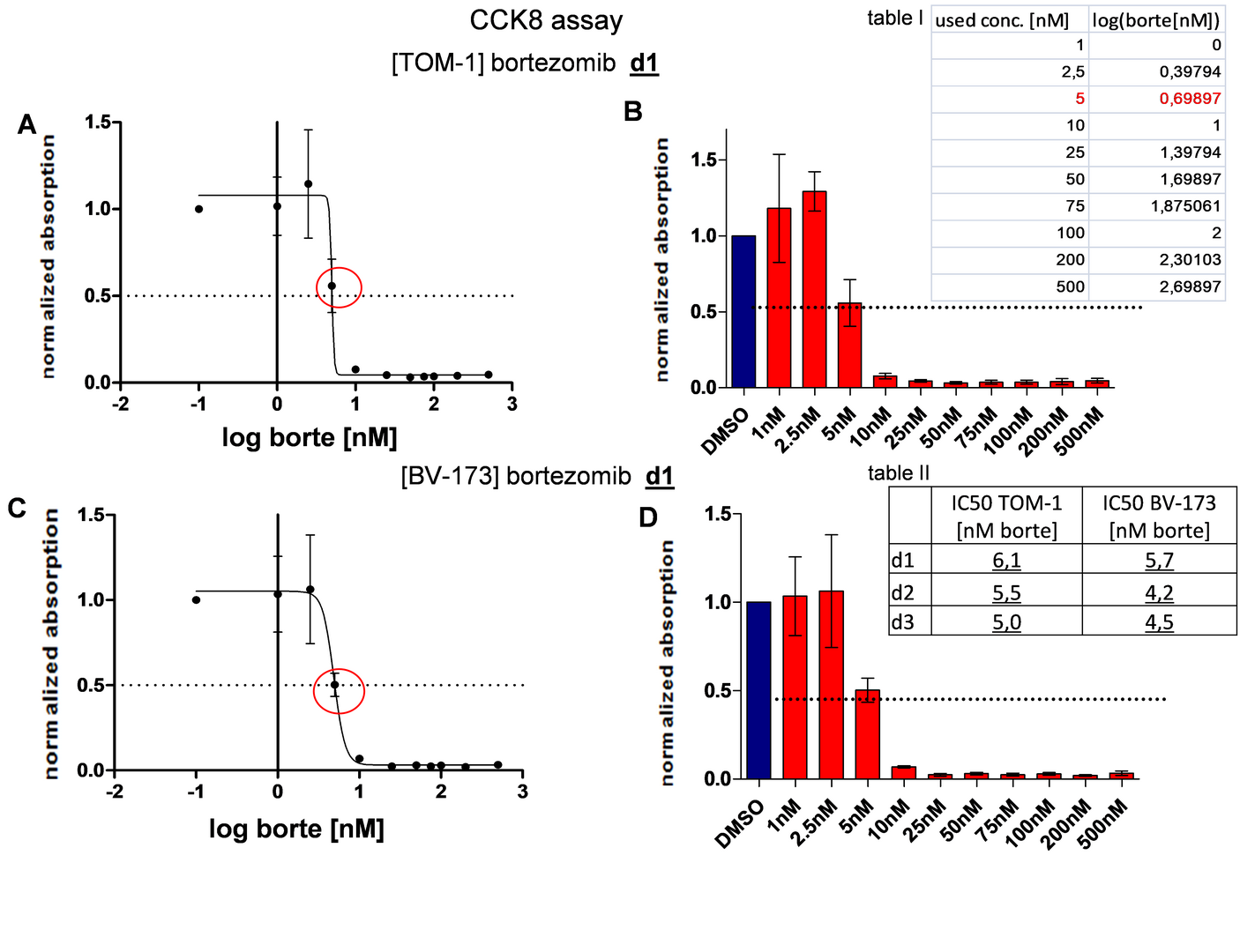

Supplement: S1 Fig — Ph+ cells BV-173 (A+B) and TOM-1 (C+D) were seeded with DMSO and bortezomib at day 0. DMSO was employed as a vehicle control in equal volume as the drug volume added in combination treatment. CCK8 assay was performed at day 1,2 and 3. The IC50 is depicted in a non-linear regression curve with the point of inflections representing the respective IC50. The drug concentration ranged from 0.001μM to 0.5μM for both drugs. The concentrations and the corresponding logarithm to construct the regression curve are shown in Table 1. Table 2: We calculated an IC50 for TOM-1 of 5nM and of 4.48nM for BV-173 on day 3. (errobars = SD, n = 2 for each treatment). (TIF) [file pone.0268352.s001.tif]

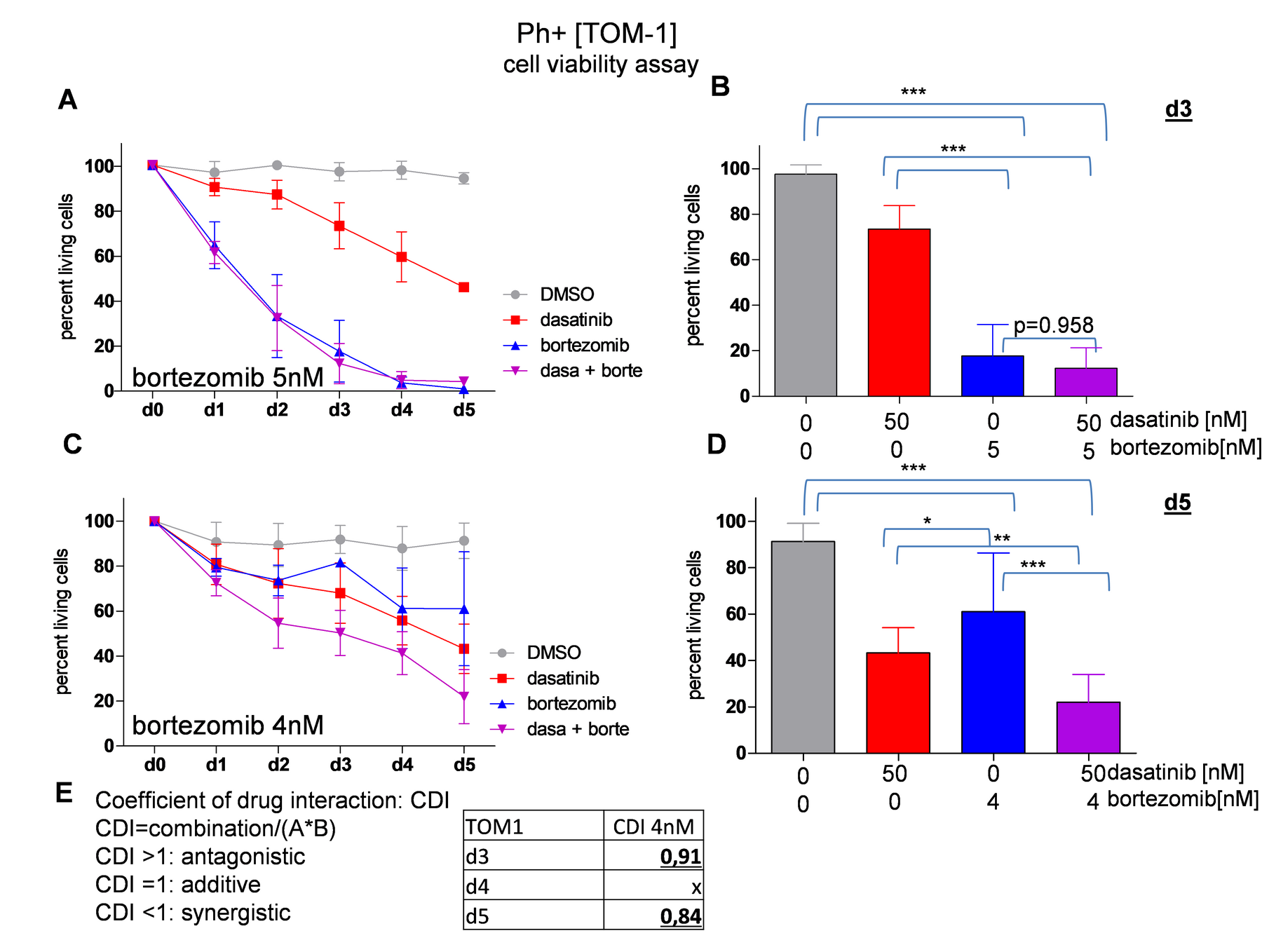

Supplement: S2 Fig — (A) Ph+ cells TOM-1 seeded with DMSO, dasatinib 50 nM, bortezomib 5 nM and the combination at day 0, drugs were replenished every third day. Viability was measured every 24h until day 5 by PI staining assay. The single as well as combined bortezomib treatment caused a massive reduction of the cell viability up to day 5. (B) Cell viability decreased to 73.49±10.2% after three days of dasatinib treatment while bortezomib treatment left only 17.78±13.7% of cells alive at this time point. (C+D) TOM-1 seeded with DMSO, dasatinib 50 nM, bortezomib 4 nM and the combination at day 0, drugs were replenished every third day. Viability was measured every 24h until day 5 by PI staining assay. At day 5 cell viability of the dasatinib treated cells decreased to 43.21±11%. Whereas the combination treatment led to only 21.97±12% viable cells. (E) Bortezomib 4nM: The calculated CDI was 0.84 on day 5, representing a synergistic effect of these two drugs in combination.(p values were calculated by one-way ANOVA with Bonferroni multiple comparison test. *,p<0.05, **,p<0.01, ***,p<0.001, error bars = SD, n = 3/3). (TIF) [file pone.0268352.s002.tif]

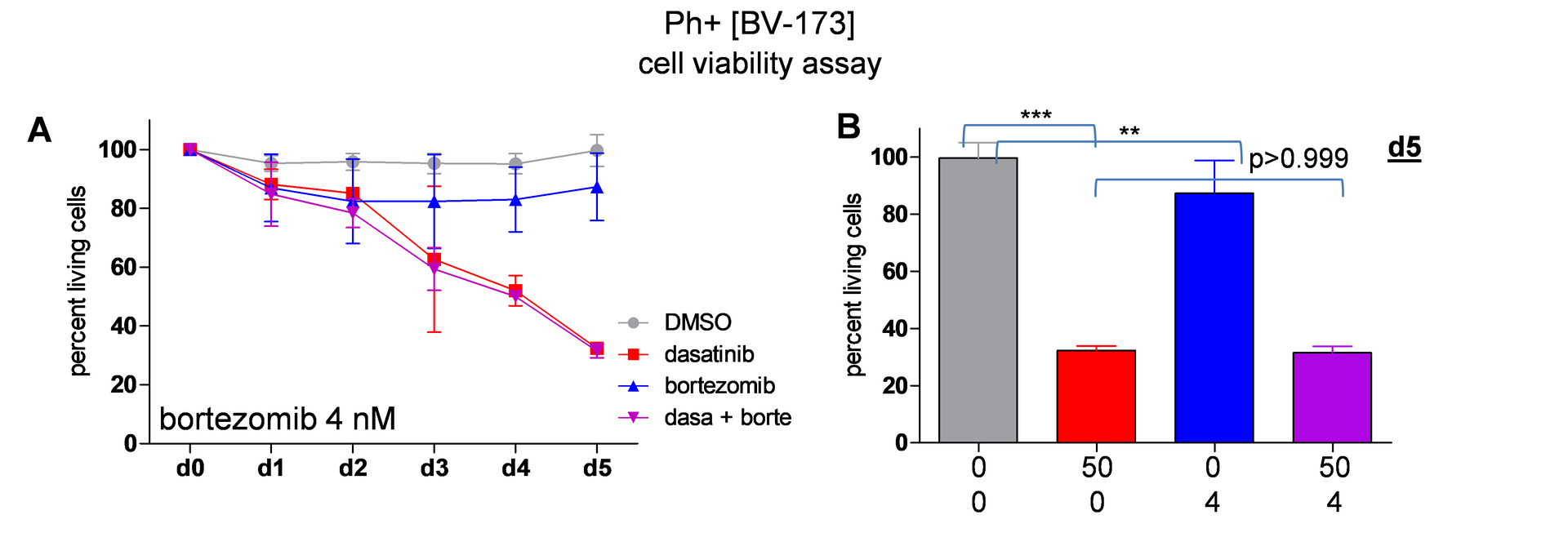

Supplement: S3 Fig — (A+B): Ph+ cells BV173 were treated with DMSO, dasatinib 50nM, bortezomib 4nM and combination at day 0. Viability was measured every 24h to day 5 using PI staining assay. Dasatinib as well as the combination of dasatinib and bortezomib caused a reduction of the viability. On day 5 32.4±1.5% of the dasatinib treated cells were viable, compared to 31.5±2.3% viability in combinated treatment. No significant different between these treatment regimes. No antagonistic effect could be detected. The strong effect of dasatinib seems to outweighs the effect of lower dose bortezomib in combination treatment with 4nM bortezomib. (p values were calculated by one-way ANOVA with Bonferroni multiple comparison test. *,p<0.05, **,p<0.01, ***,p<0.001, error bars = SD, n = 3). (TIF) [file pone.0268352.s003.tif]

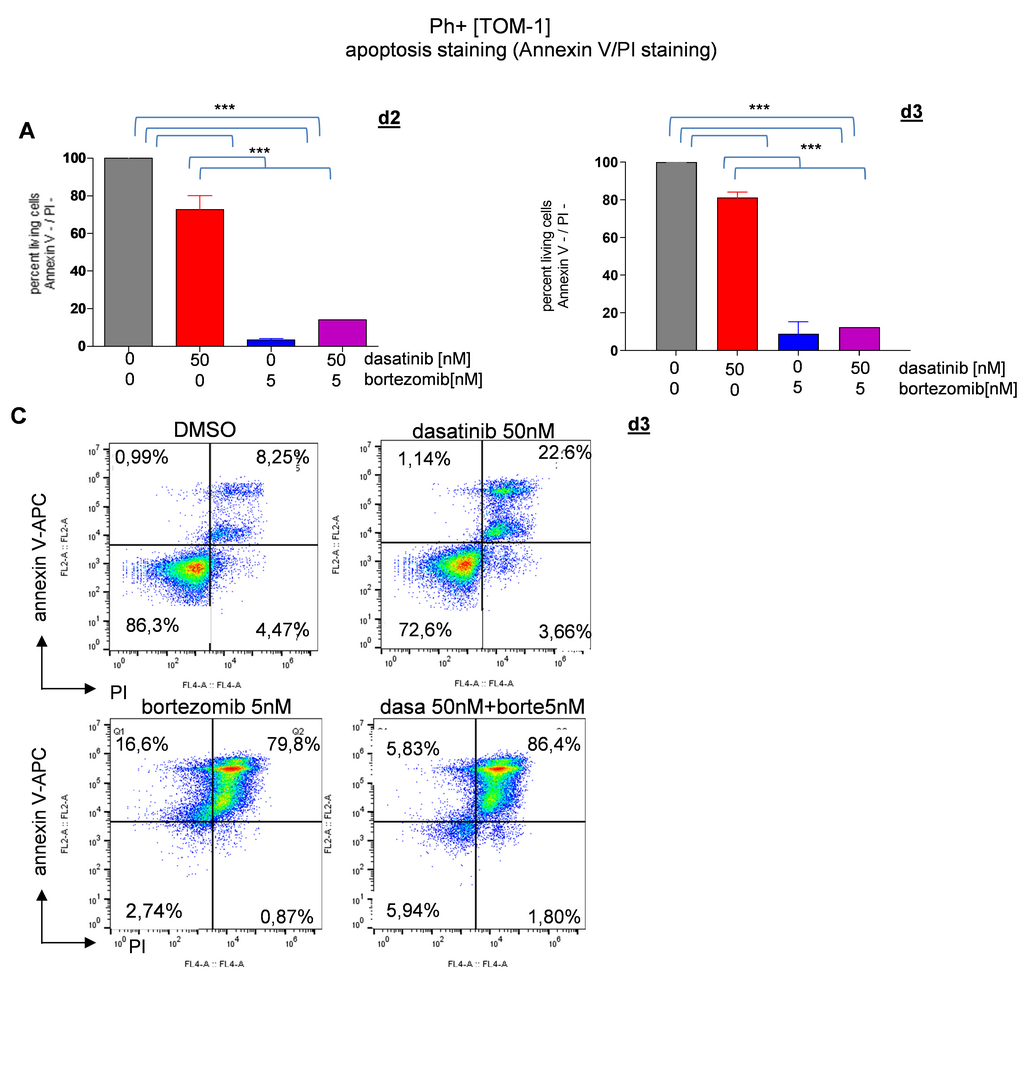

Supplement: S4 Fig — Ph+ cells TOM-1 were seeded with DMSO and bortezomib at day 0, apoptosis assay was separately performed on day 2 and day 3. Only day 3 is shown in graphics. (A+B) A significant proportion of TOM-1 cell treated with either bortezomib or the combinatorial treatment was apoptotic either at day 2 and day 3. (C) With the combination treatment of dasatinib and bortezomib, 86.4% of TOM-1 are detected in late apoptosis at day 3 (annexin V+/PI+). (p values were calculated by one-way ANOVA with Bonferroni multiple comparison test. *,p<0.05, **,p<0.01, ***,p<0.001, error bars = SD, n = 2/2). (TIF) [file pone.0268352.s004.tif]

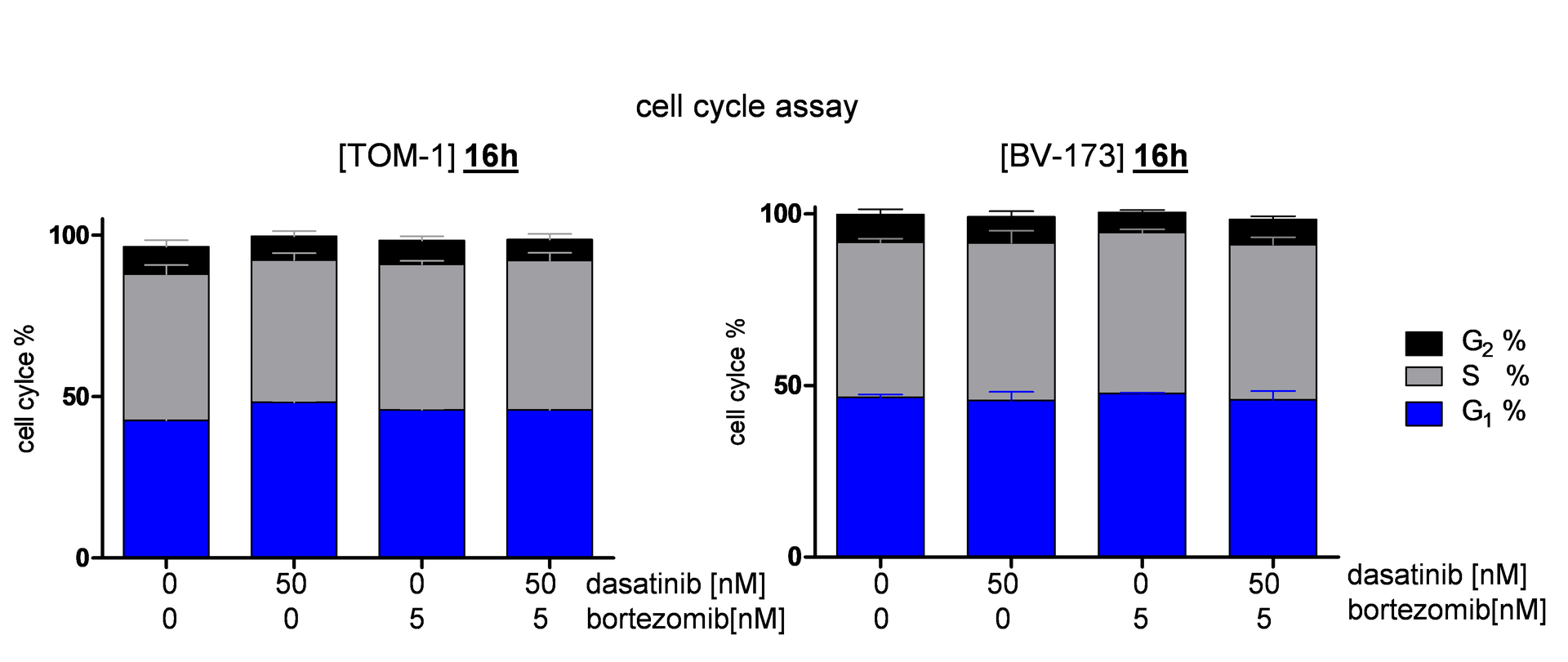

Supplement: S5 Fig — There is no significant difference in cell cycle between treatment groups, especially no significant S-phase reduction in both cell lines TOM-1 and BV-173. (error bars = SD, n = 1 for each treatment). (TIF) [file pone.0268352.s005.tif]

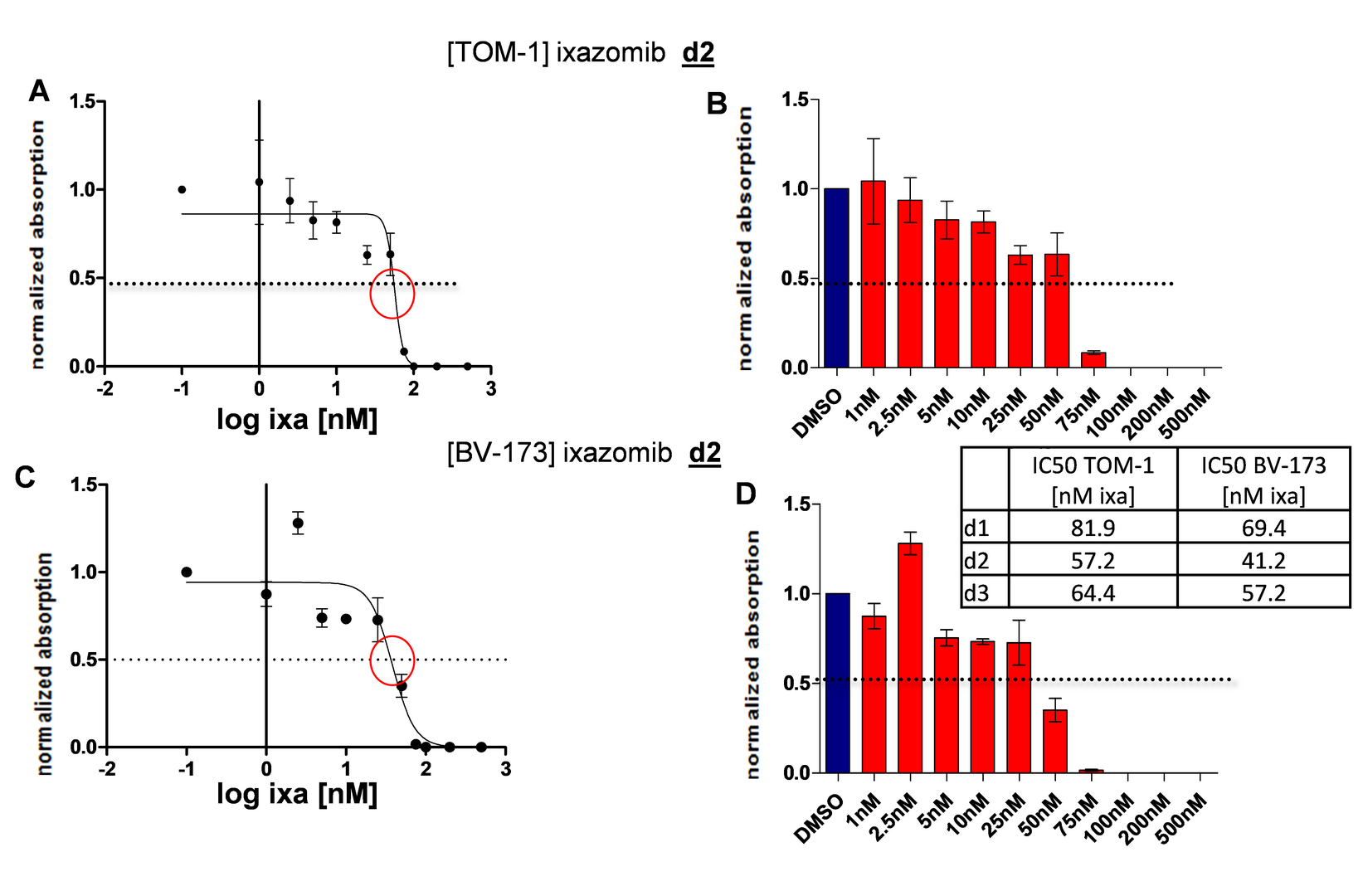

Supplement: S6 Fig — Ph+ cells BV-173 and TOM-1 were treated with DMSO and bortezomib at day 0, CCK8 assay was performed at day 1,2 and 3. The IC50 is depicted in a non-linear regression curve with the point of inflections representing the respective IC50. The calculated IC50 values are shown in the table and ranged around 57nM for TOM-1 at d2 and 41.2nM for BV-173 at day 2. Errobars = SD, n = 2 for each treatment. (TIF) [file pone.0268352.s006.tif]

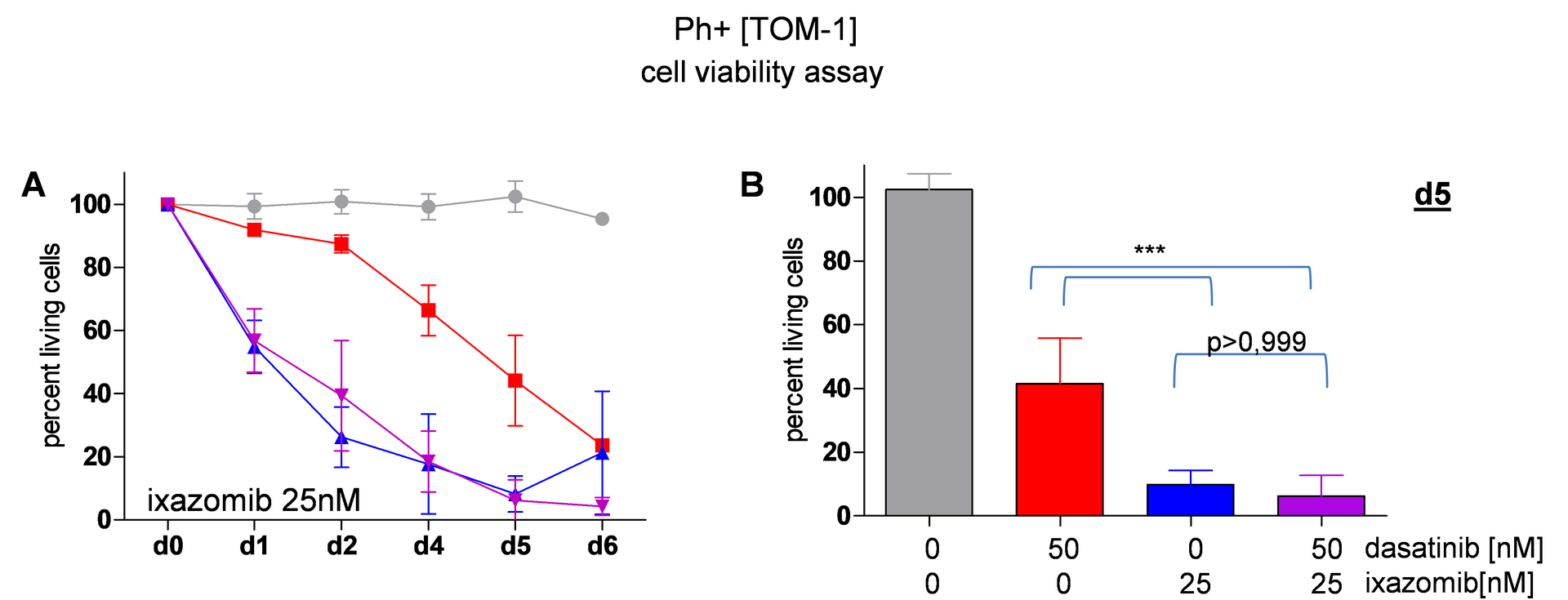

Supplement: S7 Fig — (A+B) Ph+ cells TOM-1 were treated with DMSO, dasatinib 50nM, ixazomib 25nM and the combination on day 0. Viability was measured every 24h to day 6 using PI staining assay. Drugs were replenished every third day. Ixazomib and combination treatment lead to a significant reduction of the viability. At day 5 only 6.2±2.9% of the combinated treated cells were viable. No synergistic activity between ixazomib and dasatinib as well as no antagonistic effect. (p values were calculated by one-way ANOVA with Bonferroni multiple comparison test. *,p<0.05, **,p<0.01, ***,p<0.001, error bars = SD, n = 3). (TIF) [file pone.0268352.s007.tif]

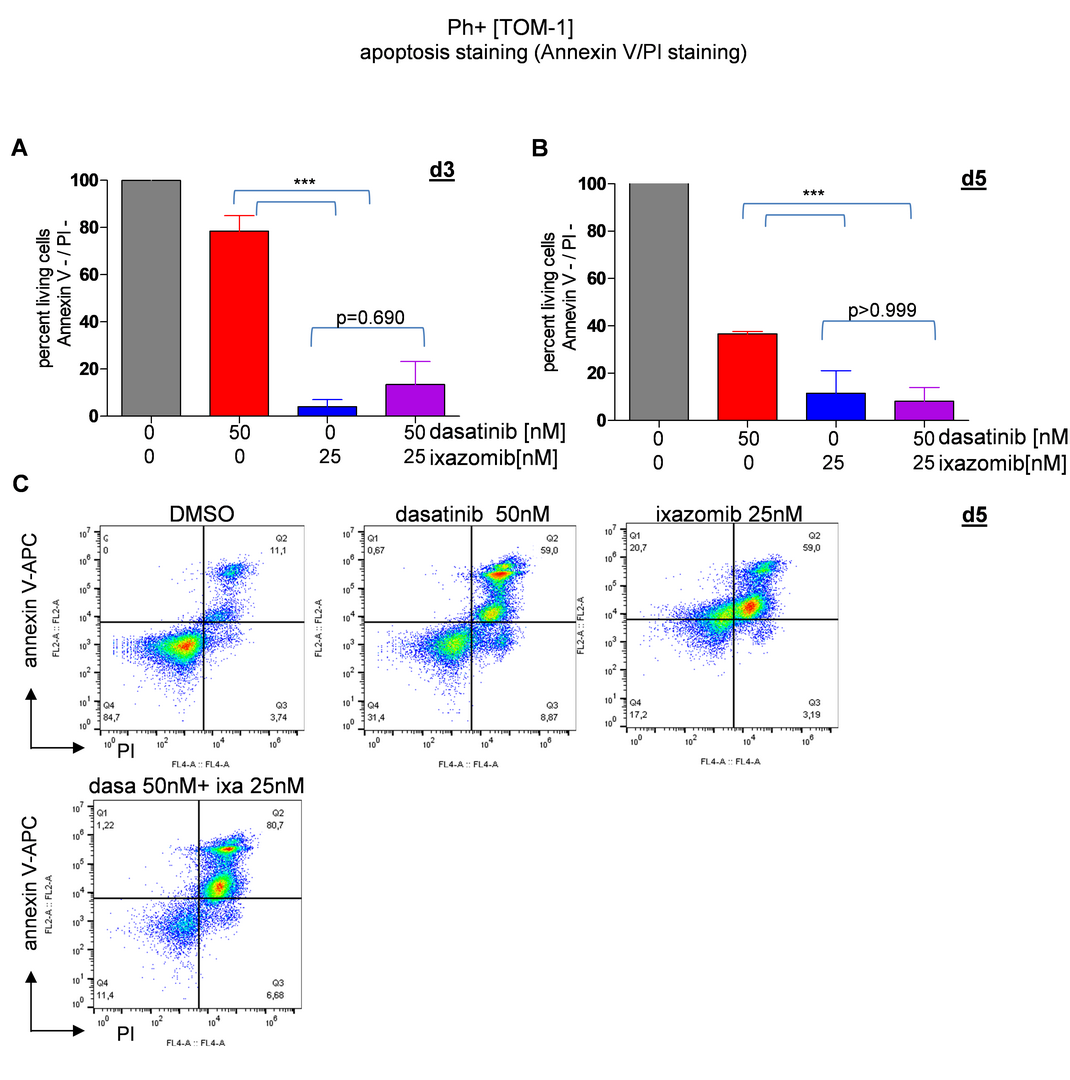

Supplement: S8 Fig — Ph+ cells TOM-1 were seeded with DMSO, dasatinib 50nM, ixazomib 25nM and the combination of both at day 0, apoptosis assay was performed at day 3 and day 5. (A) At day 3 13.4±9.7% of the cells treated with ixazomib 25nM + dasatinib were non apoptotic (annexin V-/PI-). (B) At day 5 8.1±5.8% of the cells treated with ixazomib 25nM + dasatinib were non apoptotic (annexin V-/PI-). In the graphics only day 5 is shown. (C) 80.7% in late apoptosis at day 5 (annexin V+/PI+) with the dual treatment (ixazomib 25nM + dasatinib) and 6.6% in early apoptosis. For all analyses, p values were calculated by one-way ANOVA with Bonferroni multiple comparison test. A p value of less than 0.05 was considered statistically significant for all analyses. (*,p<0.05, **,p<0.01, ***,p<0.001), error bars = SD, n = 2/2). (TIF) [file pone.0268352.s008.tif]

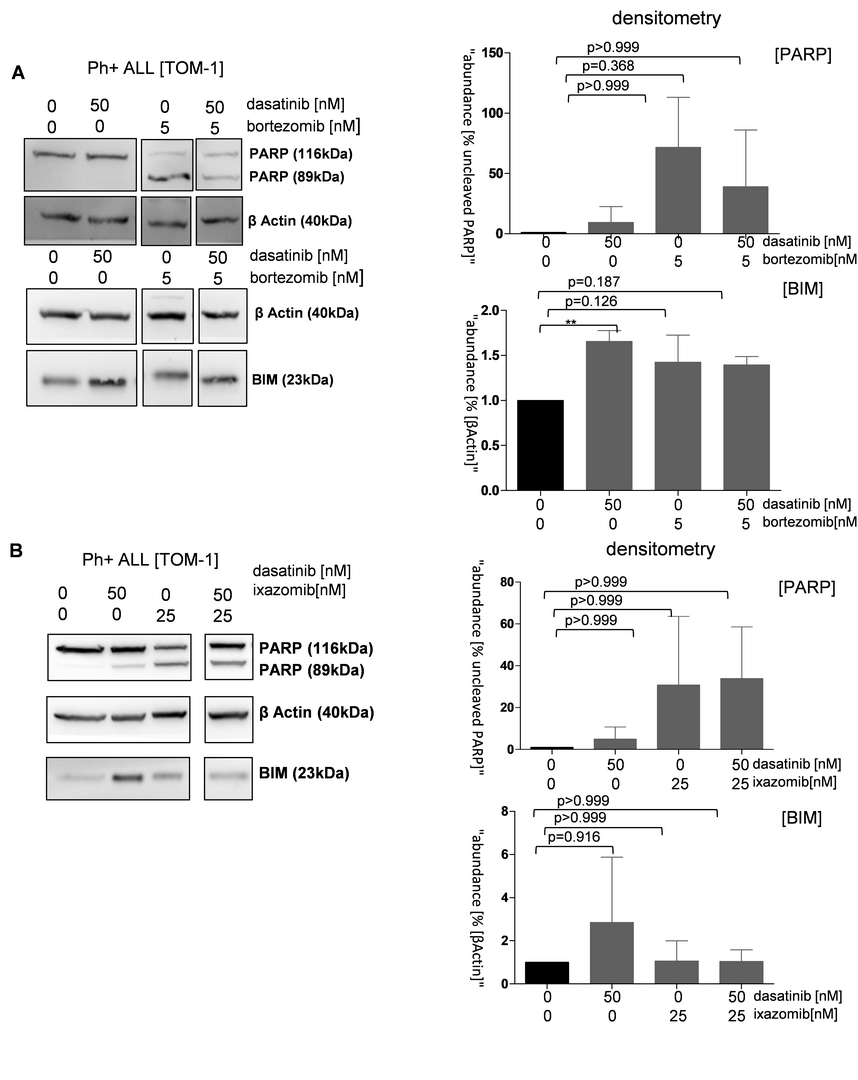

Supplement: S9 Fig — TOM-1 cell were treated for 16 hours with dasatinib 50nM, proteasome inhibitor bortezomib and ixazomib and the combination of both drugs. Proteasome inhibitor treatment causes an upregulation of cleaved PARP in all treated cells. TKI treatment upregulates proapoptotic BIM. Densitometry analysis was performed using ImageJ® software and also showed an upregulation of PARP-1 under proteasome inhibitor therapy and an increased expression of BIM by dasatinib administration. These results were not statistically significant. (p values were calculated by one-way ANOVA. *,p<0.05, **,p<0.01, ***,p<0.001, error bars = SD, n = 2/3/3/3). (TIF) [file pone.0268352.s009.tif]

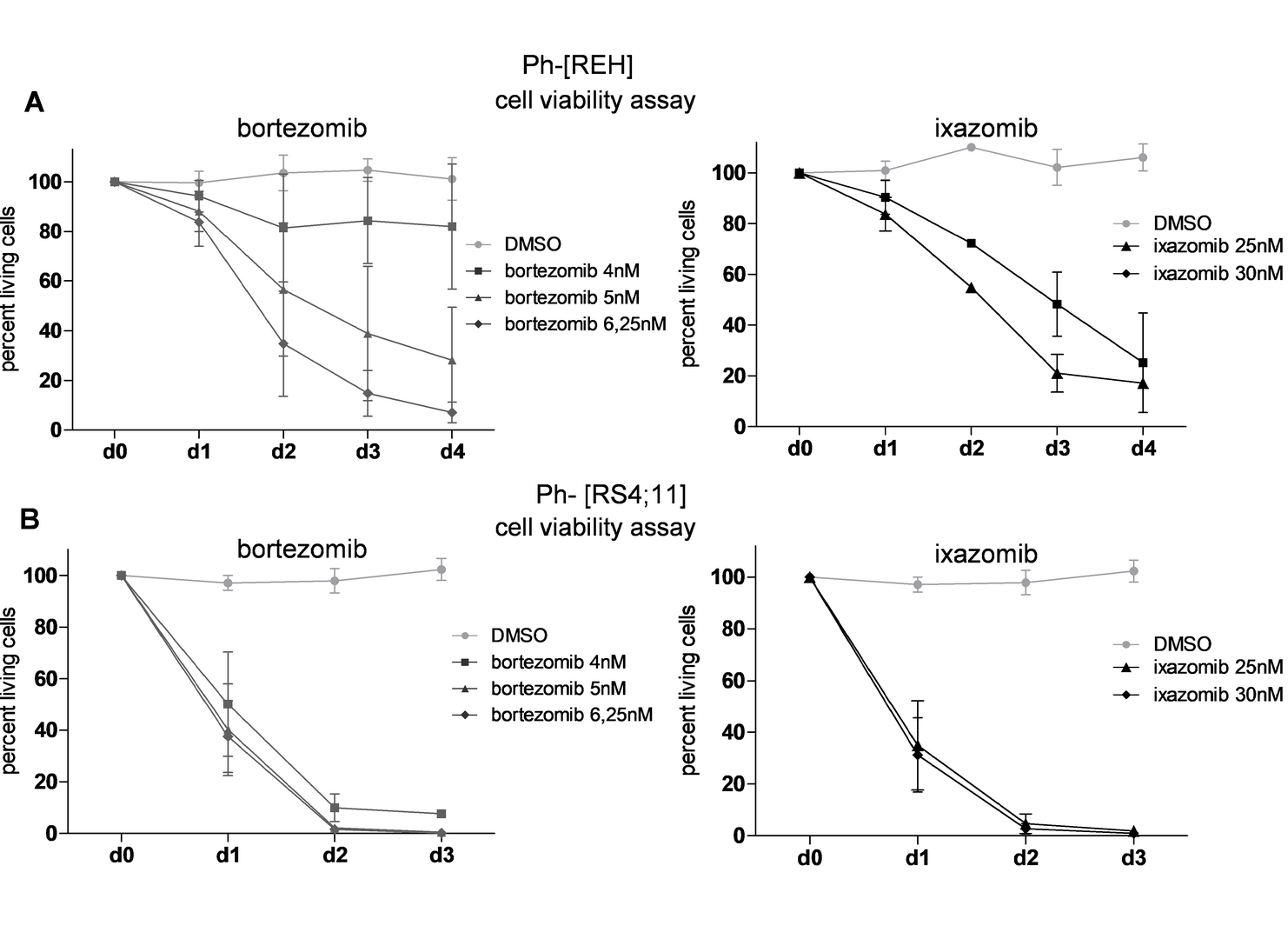

Supplement: S10 Fig — Ph- ALL cells REH and RS4;11 were seeded with DMSO, bortezomib 4nM, 5nM, 6.25nM and ixazomib 25nM, 30nM at day 0, drugs were replenished every third day. Viability was measured every 24h to day 3/4 using PI staining assay. Treatment with bortezomib and ixazomib causes a significant (***) reduction of viability in the two Ph- ALL cell lines REH and RS4;11. At d314.84±9.18% of the REH cells treated with bortezomib 5nM were viable and only 21.01±7.43% treated with ixazomib treatment were alive. For the RS4;11 cells, only 0.16±0.03% of the cells treated with bortezomib were viable at day 3. Similar effects were caused by ixazomib 30nM treatment: only 1±0.94% of the treated cells were alive at day 3. (p values were calculated by one-way ANOVA with Bonferroni multiple comparison test. *,p<0.05, **,p<0.01, ***,p<0.001, error bars = SD, n = 3 for each treatment). (TIF) [file pone.0268352.s010.tif]
