# Supplementary material for: Combined inhibition of BCR-ABL1 and the proteasome as a potential novel therapeutic approach in BCR-ABL positive acute lymphoblastic leukemia
Source: PLoS One. 2022 Oct 4;17(10):e0268352. doi: 10.1371/journal.pone.0268352 (PMC9531817; doi:10.1371/journal.pone.0268352)

# Westernblot original pictures

10.04.18 Western-blot TOM1 treated with DMSO, dasatinib 50nM, bortezomib 4nM, 5nM, dasa+borte

timepoints: 16h

Ph+ ALL [TOM1]

| | | | | | | |
|---|---|---|---|---|---|---|
| 0 | 50 | 0 | 0 | 50 | 50 | dasatinib [nM] |
| 0 | 0 | 4 | 5 | 4 | 5 | bortezomib[nM] |

180kDa
130kDa
100kDa
70kDa

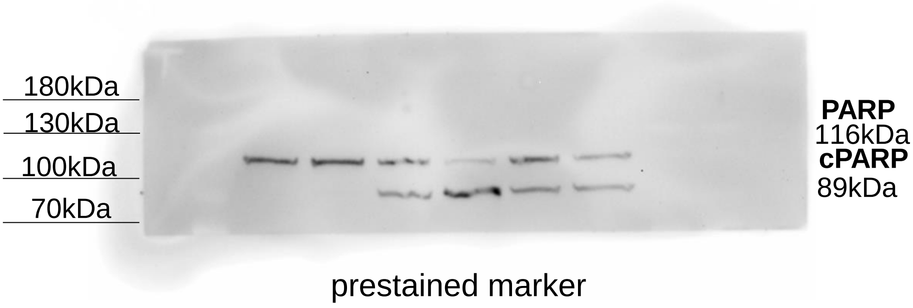

**PARP**
116kDa
**cPARP**
89kDa

prestained marker

180kDa
130kDa
100kDa
70kDa

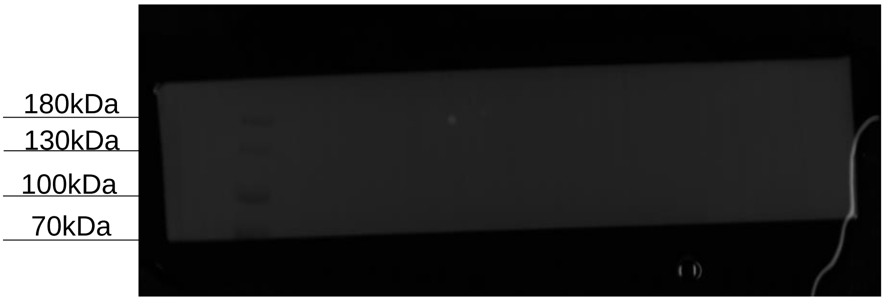

| | | | | | |
|---|---|---|---|---|---|
| 0 | 50 | 0 | 0 | 50 | 50 |
| 0 | 0 | 4 | 5 | 4 | 5 |

70kDa
55kDa
40kDa

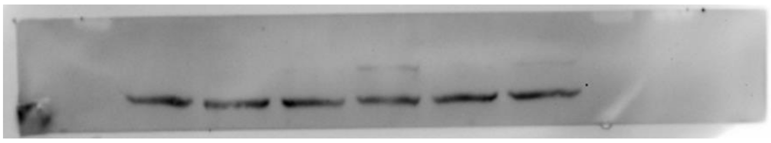

**β Actin (42kDa)**

prestained marker

70kDa
55kDa
40kDa

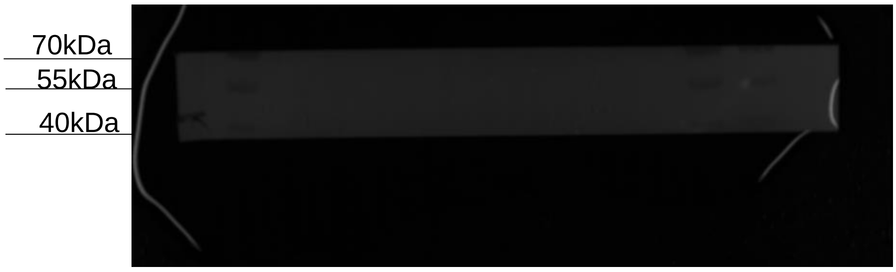

Figure no Fig3 in the manuscript

Prestained marker: Thermo Scientific™ PageRuler™ Prestained Protein Ladder, 10 to 180 kDa

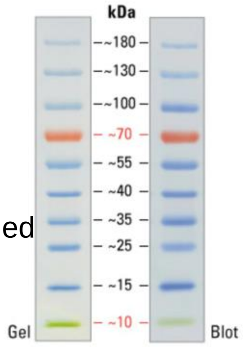

29.03.18 Western-blot TOM1 treated with DMSO, dasatinib 50nM, bortezomib 4nM, 5nM, dasa+borte
timepoints: 16h

Ph+ ALL [TOM1]

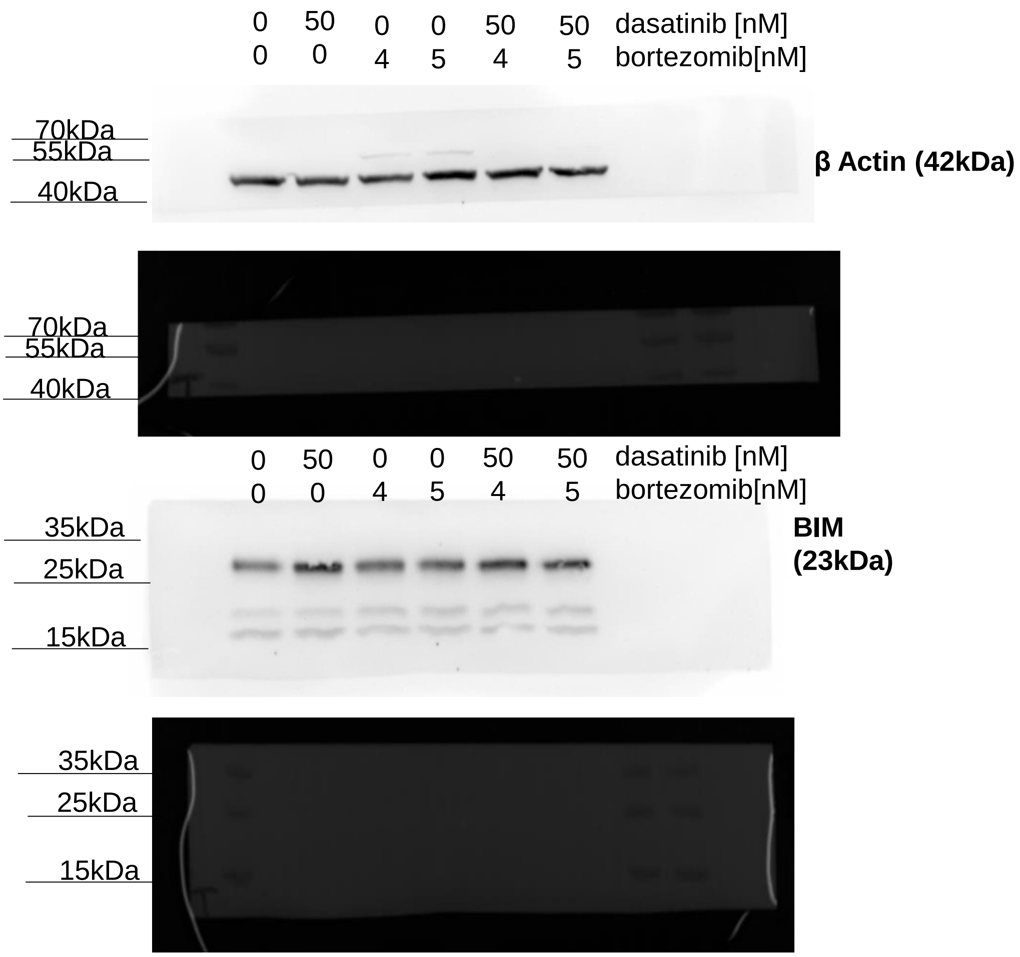


Figure no Fig3. in the manuscript

Prestained marker: Thermo Scientific™ PageRuler™ Prestained Protein Ladder, 10 to 180 kDa

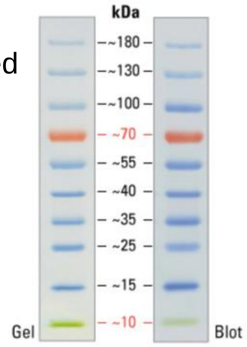

03.11.18 Western-blot BV173 treated with DMSO, dasatinib 50nMl ixazomib 25nM, 30nM, dasa + ixa
Timepoints: 16h

Ph+ ALL [BV-173]

| | | | | | | |
|---|---|---|---|---|---|---|
| 0 | 50 | 0 | 0 | 50 | 50 | dasatinib [nM] |
| 0 | 0 | 25 | 30 | 25 | 30 | ixazomib[nM] |

180kDa
130kDa
100kDa
70kDa

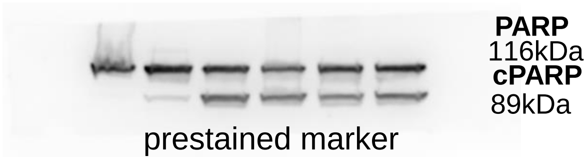

**PARP**
116kDa
**cPARP**
89kDa

prestained marker

180kDa
130kDa
100kDa
70kDa

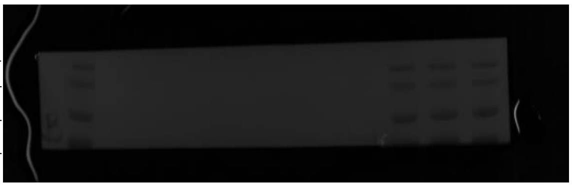

| | | | | | | |
|---|---|---|---|---|---|---|
| 0 | 50 | 0 | 0 | 50 | 50 | dasatinib [nM] |
| 0 | 0 | 25 | 30 | 25 | 30 | ixazomib[nM] |

70kDa
55kDa
40kDa

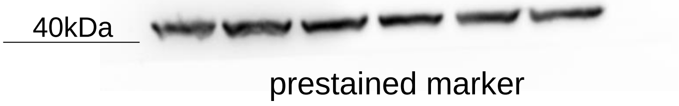

**β Actin (42kDa)**

prestained marker

70kDa
55kDa
40kDa

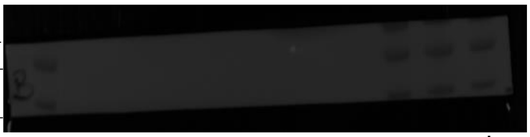

| | | | | | | |
|---|---|---|---|---|---|---|
| 0 | 50 | 0 | 0 | 50 | 50 | dasatinib [nM] |
| 0 | 0 | 25 | 30 | 25 | 30 | ixazomib[nM] |

25kDa
15kDa

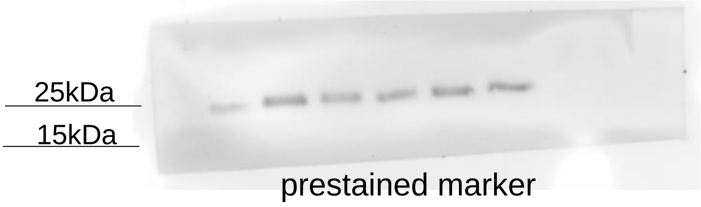

**BIM (23kDa)**

prestained marker

25kDa
15kDa

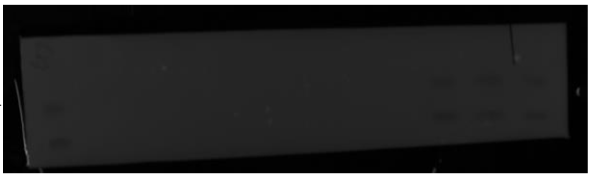

Figure no Fig3. in the manuscript

Prestained marker: Thermo Scientific™ PageRuler™ Prestained Protein Ladder, 10 to 180 kDa

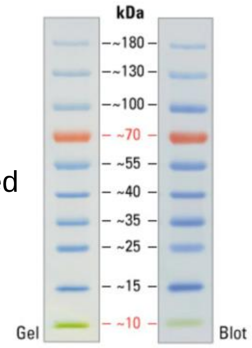

10.04.18 Western-blot  BV173 treated with DMSO, Dasatinib 50nM, Bortezomib 6,25nM, Dasa+Borte
Timepoints: 16h

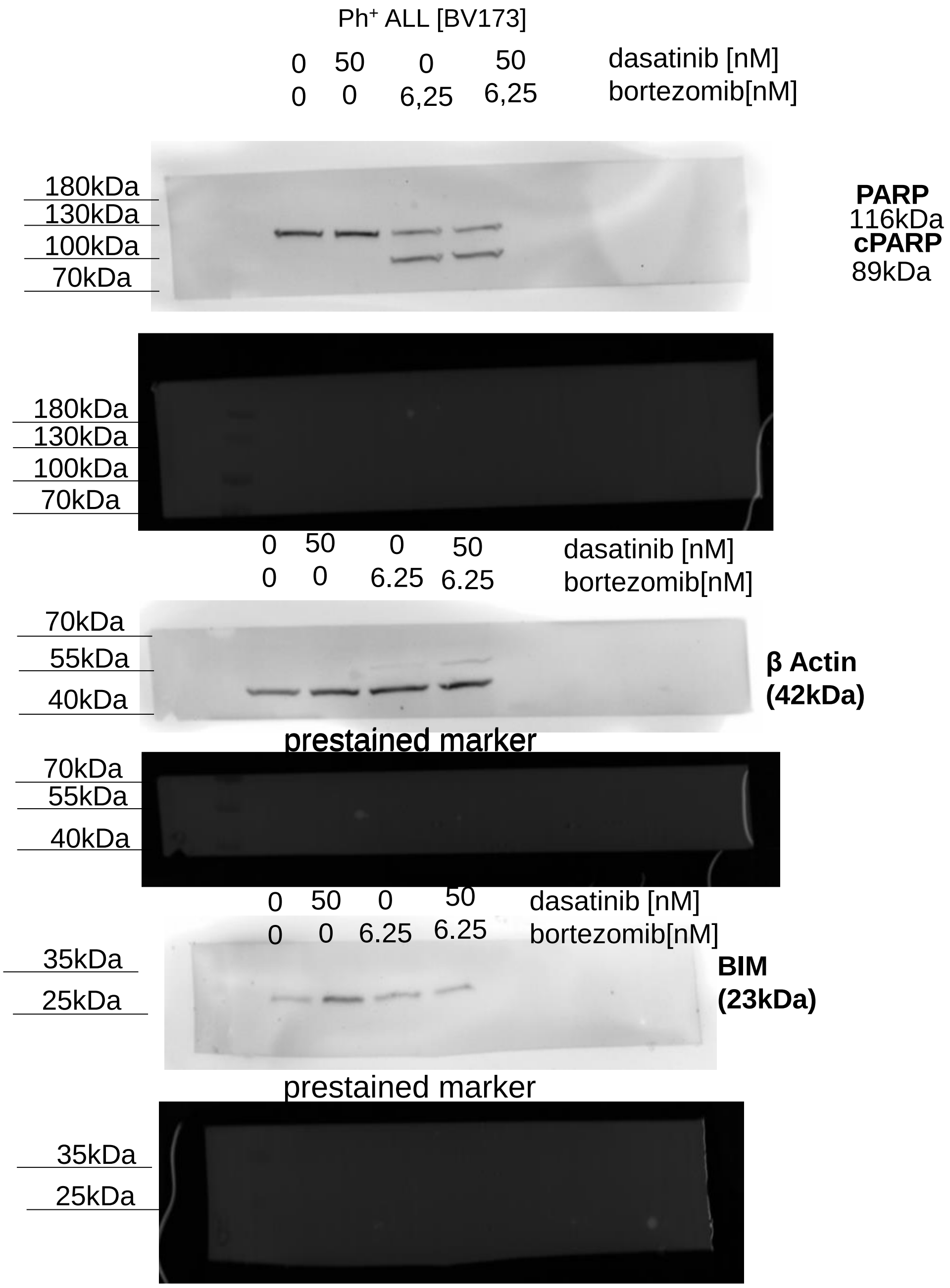


Figure no  SFig8. in the manuscript

Prestained marker: Thermo Scientific™ PageRuler™ Prestained Protein Ladder, 10 to 180 kDa

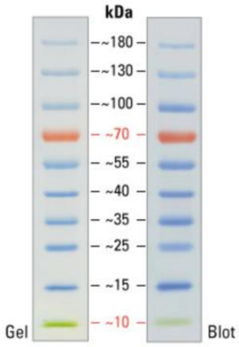

03.11.18 Western-blot  TOM1 treated with DMSO, dasatinib 50nM, ixazomib 25nM, 30nM, dasa + ixa

timepoints: 16h

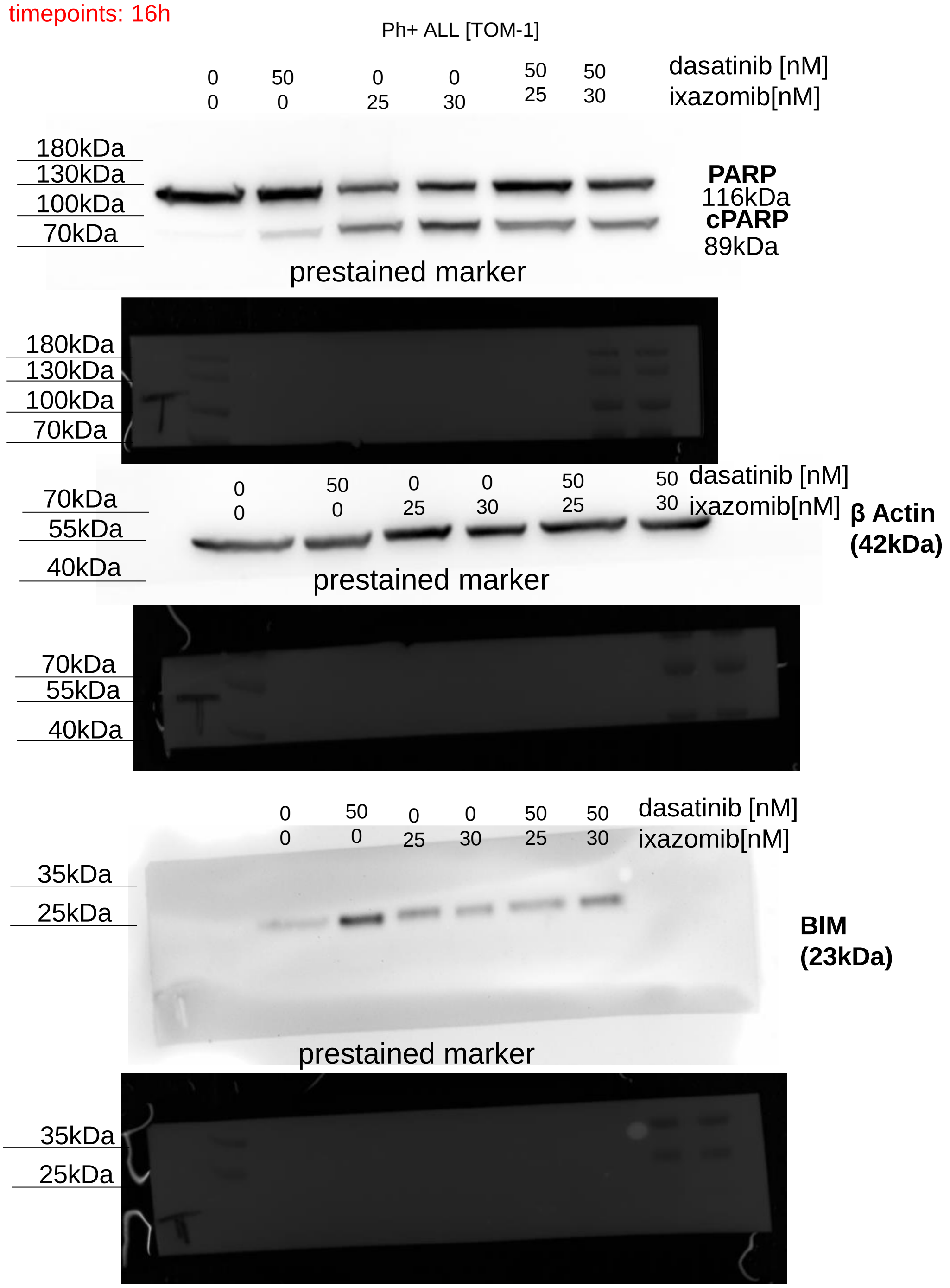


Figure no  SFig8. in the manuscript

Prestained marker: Thermo Scientific™ PageRuler™ Prestained Protein Ladder, 10 to 180 kDa

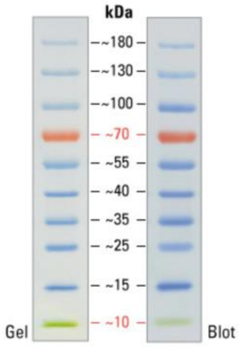

Supplement: S1 Raw images — (PDF) [file pone.0268352.s011.pdf]
